# Single-cell RNA sequencing reveals the immune features and viral tropism in the central nervous system of mice infected with Japanese encephalitis virus

**DOI:** 10.1186/s12974-024-03071-1

**Published:** 2024-03-26

**Authors:** Ling’en Yang, Junyao Xiong, Yixin Liu, Yinguang Liu, Xugang Wang, Youhui Si, Bibo Zhu, Huanchun Chen, Shengbo Cao, Jing Ye

**Affiliations:** 1 National Key Laboratory of Agricultural Microbiology, Huazhong Agricultural University, Wuhan, 430070 Hubei China; 2 Frontiers Science Center for Animal Breeding and Sustainable Production, College of Veterinary Medicine, Huazhong Agricultural University, Wuhan, 430070 Hubei People’s Republic of China; 3 Hubei Hongshan Laboratory, Wuhan, 430070 Hubei People’s Republic of China; 4 The Cooperative Innovation Center for Sustainable Pig Production, Huazhong Agricultural University, Wuhan, Hubei China

**Keywords:** JEV, scRNA-seq, Neuron, Baiap2, Inflammation

## Abstract

Japanese encephalitis virus (JEV) is a neurotropic pathogen that causes lethal encephalitis. The high susceptibility and massive proliferation of JEV in neurons lead to extensive neuronal damage and inflammation within the central nervous system. Despite extensive research on JEV pathogenesis, the effect of JEV on the cellular composition and viral tropism towards distinct neuronal subtypes in the brain is still not well comprehended. To address these issues, we performed single-cell RNA sequencing (scRNA-seq) on cells isolated from the JEV-highly infected regions of mouse brain. We obtained 88,000 single cells and identified 34 clusters representing 10 major cell types. The scRNA-seq results revealed an increasing amount of activated microglia cells and infiltrating immune cells, including monocytes & macrophages, T cells, and natural killer cells, which were associated with the severity of symptoms. Additionally, we observed enhanced communication between individual cells and significant ligand-receptor pairs related to tight junctions, chemokines and antigen-presenting molecules upon JEV infection, suggesting an upregulation of endothelial permeability, inflammation and antiviral response. Moreover, we identified that Baiap2-positive neurons were highly susceptible to JEV. Our findings provide valuable clues for understanding the mechanism of JEV induced neuro-damage and inflammation as well as developing therapies for Japanese encephalitis.

**Supplementary Information:**

The online version contains supplementary material available at 10.1186/s12974-024-03071-1.

## Background

In recent years, there has been an increase in the frequency of outbreaks of neurotropic infectious diseases worldwide, including Rift Valley fever, West Nile fever, and Zika. These outbreaks pose significant risks to both human and animal life [1]. Japanese encephalitis virus (JEV) is a typical neurotropic pathogen that triggers massive inflammatory response in the central nervous system (CNS). It is highly prevalent in Central Asia and Southeast Asia, characterized by high mortality and disability rates [2, 3]. Despite the use of various vaccines to control Japanese encephalitis [4], it is alarming that 80% of cases still occur in areas where Japanese encephalitis vaccination plans are implemented [5]. Unfortunately, there is currently no specific treatment available for JEV infection. Hence, development of new therapeutic methods and drugs is of utmost importance.

The efficient infection and proliferation of JEV on neurons result in neuronal damage and death. This neuronal damage also triggers the activation of glial cells, which release large amounts of inflammatory factors, further exacerbating the neuronal damage [6–11]. Additionally, JEV can directly infect glial cells, leading to their activation and initiating the inflammatory response [12–14]. Upon JEV infection in CNS, the host cells identify pathogen associated molecular patterns (PAMPs) through pattern recognition receptors (PRRs). This recognition process activates the innate immune system, initiating a signaling cascade that results in elevated levels of interferon, inflammatory cytokines, and chemokines [15, 16]. For instance, JEV infection could induce the activation of inflammatory response in glial cells through the recognition of TLR3 and RIG-I [17]. In addition, c-Jun N-terminal kinase 1(JNK1) was identified as a key signaling molecule for activation of glial cells and induction of inflammatory responses during JEV infection [18]. The high permeability of neurons to JEV plays a crucial role in JEV-induced neuronal damage and inflammation in CNS. Recent studies have implicated the role of glucose-regulated protein 78 (GRP78), a member of the heat shock protein 70 (HSP70) family, in JEV entry into Neuro2a and BHK-21 cells [19, 20]. Apart from HSP70 and GRP78, heat shock protein 90 Beta (HSP90β) has also been found to interact with the JEV envelope (E) protein and may facilitate JEV entry into mammalian cells [21]. Another member of the HSP70 family, heat shock cognate protein 70 (HSC70), has been proposed as a receptor for JEV entry into mosquito cells [22]. Bulk RNA-seq of JEV-infected cells and tissues suggests that JEV infection causes significant changes of mRNA expression profiles which associated with antiviral response of host [23, 24]. Although bulk RNA-seq analysis has provided insights into the underlying mechanism of neuronal damage and inflammation caused by JEV [24, 25], it has limited resolution in capturing the overall changes in the brain following infection. This limitation can obscure the intricate intercellular and intracellular changes, particularly in minority cell types. Consequently, interpreting the disease becomes more challenging.

The CNS is composed of various types of cells, including neurons, microglia, astrocytes, oligodendrocytes, endothelial cells and mural cells. Among these cells, neurons are the major component and have a complex structure. Based on the neurotransmitters released by neurons, they can be classified into different types such as cholinergic neurons, dopaminergic neurons, GABAergic neurons, glutamatergic neurons, and 5-hydroxytryptaminergic neurons. Additionally, they can also be categorized based on their functions as sensory neurons, motor neurons, and intermediate neurons. Recent studies have shown that the rabies virus (RABV) can invade neuronal cells by utilizing the metabotropic glutamate receptor (mGluR2) as its receptor protein, which is highly expressed in the CNS [26]. This finding provides valuable insights into the molecular mechanism of neurotropism of the virus. Another neurotropic virus, the porcine hemagglutinating encephalomyelitis virus (PHEV), has been found to bind neural cell adhesion molecules (NCAM) to invade cells [27]. In addition, mosquitoes, which are the primary transmission vectors of flaviviruses, can exploit the GABAergic system to suppress natural immunity and facilitate virus infection [28]. Previous studies have demonstrated that JEV primarily targets specific regions of the CNS, such as the cerebral cortex, striatum, and thalamus [29], indicating that the distribution of JEV in the CNS is not random but rather selective. However, it remains unclear which specific types of neurons are more susceptible to JEV and in which neuronal cells it can proliferate more extensively. Investigating the preferred neuronal types for JEV not only aids in understanding the pathogenic mechanisms of JEV and other neurotrophic pathogens, but also lays the foundation for the development of new drugs.

The single-cell RNA sequencing (scRNA-seq) is an emerging technology that provides a powerful tool for identifying cell types and cell states by analyzing the expression profile of an individual cell's transcriptome [30, 31]. This technique has been successfully utilized to gain insights into the diversity of various tissue cells, including those in the brain [32–34]. Furthermore, scRNA-seq has been applied to study a range of viral infections, such as SARS-CoV-2, RABV, influenza virus, human immunodeficiency virus (HIV), hepatitis B virus (HBV), Ebola virus, and Zika virus (ZIKV) [35–41]. These studies have provided unbiased and comprehensive visualizations of the specific cells targeted by these viruses, as well as a holistic understanding of the host's immune responses.

In this study, we conducted scRNA-seq analysis to investigate the intricate landscapes and cell–cell interactions in the brains of JEV-infected mice exhibiting different symptoms. We isolated a total of 88,000 single cells from different regions of the brain, including the cerebral cortex, striatum, and thalamus, all of which exhibited high levels of JEV infection. This allowed us to create a comprehensive atlas of the entire brain in JEV-infected mice. Our findings highlight the decreased number of neurons and increased presence of activated microglia cells and infiltrating immune cells, such as astrocytes, T cells, and NK cells, in the brain of JEV-infected mice. Additionally, we found that Baiap2 positive neurons are highly susceptible to JEV infection. Overall, our study provides a comprehensive understanding of cell landscapes and viral tropism in the brain of JEV-infected mice. This knowledge is valuable for clarifying pathogenesis of JEV and developing therapies to mitigate the burden of virus-associated encephalitis in humans.

## Materials and methods

### Mice, cell lines and virus

Female C57BL/6 mice, aged 5 to 6 weeks, were obtained from the Animal Center of Huazhong Agricultural University. All mouse experiments were conducted in accordance with the approved protocols of the Animal Care and Ethics Committee of Huazhong Agricultural University, under the reference number HZAUMO-2023-0050. The protocols followed the guidelines outlined in the Guide for the Care and Use of Laboratory Animals. BHK-21 (Baby hamster kidney) cells were cultured in dulbecco's modified eagle medium (DMEM) (Sigma-Aldrich, USA), supplemented with 10% fetal bovine serum (FBS, Every Green, China), 100 IU/mL penicillin, and 100 μg/mL streptomycin at 37 ℃ with 5% CO2. The JEV-P3 strain virus was stored in our laboratory.

### Animal infection

Female C57BL/6 mice, aged 5 to 6 weeks and weighing approximately 16 g, were intraperitoneally injected with 10^5^ PFU of JEV P3 strain (100 μL) or same volume of DMEM (Sigma-Aldrich, USA). The health condition and weight change of mice were monitored daily. On the 7dpi, three mock-infected mice and five JEV-infected mice exhibiting different symptoms were selected for single cell sequencing.

### Viral plaque assay

The brain tissue suspensions of JEV-infected mice were serially tenfold diluted in DMEM and incubated with BHK-21 cells in 24-well plates at 37 °C for 1 h. After incubation, the supernatants were removed and the cells were washed three times with serum-free DMEM. Subsequently, the cells were incubated for 4 days in DMEM containing 2% FBS and 1.5% sodium carboxymethyl cellulose at 37 °C in an incubator. Finally, the cells were fixed with 10% formaldehyde and stained with 0.6% crystal violet solution. Viral titers were calculated based on visible plaques.

### Single cell isolation

Mice were anesthetized by injection of ketamine hydrochloride and then perfused through the heart with ice-cold Choline Chloride Solution (CCS) containing the following components (in mM): 92 Choline chloride, 2.5 KCl, 1.2 NaH_2_PO_4_, 30 NaHCO_3_, 20 HEPES, 25 Glucose, 5 Sodium ascorbate, 2 Thiourea, 3 Sodium pyruvate, 10 MgSO_4_7H_2_O, 0.5 CaCl_2_2H_2_O, 12 N-Acetyl-L-cysteine (pH = 7.4, Osmotic pressure = 310). The brains were then removed and placed in a slicing chamber containing ice-cold “CCS + Inhibitor buffer”, which consists of CCS with 10 μM NBQX (Apexbio, B6566) and 50 μM APV (MedChemExpress, HY-100714A). Brain slabs, approximately 300 μm thick, were cut using a Leica VT 1200S vibratome. Slabs containing the regions of the cerebral cortex, striatum, and thalamus were gently isolated under a stereoscope and transferred into a 15 mL Falcon tube containing 5 mL of “CCS + Inhibitor buffer”. They were then incubated for 30 min at 33 ℃ before being transferred into 2 mL of “CCS + Enzyme I Buffer” in a 15 mL Falcon tube. The “CCS + Enzyme I Buffer” was activated at 37 °C for 15 min before use. The “CCS + Enzyme I Buffer” consists of CCS with 20 units/mL Papain (Worthington, LS003126) and 100 units/mL DNase I (Sigma, D4527-40KU). Digestion was performed at 37 °C for 20 min, with mixing every 5 min. The brain tissue was digested in 2 mL of “CCS + Enzyme II Buffer”, which consists of CCS with 20 mg/mL Protease (Sigma, P5147-1G), 20 mg/mL Dispase (Worthington, LS02106), and 100 units/mL DNase I (Sigma, D4527-40KU). Digestion was performed on a shaker at 25 °C for 20 min. Tubes containing the digested tissue were transferred onto ice, and the “CCS + Enzyme II Buffer” was replaced with 2 mL of “CCS + Stop buffer” containing CCS and 2 mg/mL BSA (Sigma-Aldrich, A2153) and 0.2 units/mL DNase I. Tissue chunks were then carefully titrated with a series of n = 5 fire-polished pasteur pipettes with successively smaller bores. Bubbles were avoided, and 1 mL of supernatant was aspirated with each blow of tissue, followed by the addition of 1 mL of buffer. Falcon tubes containing 10 mL of titrated cells were then filtered by 40 μm filtration and centrifuged at 500*g* for 5 min. The supernatant was removed and the pellet was then resuspended in 3.1 mL of “CCS + Stop buffer” in a new tube. It was mixed with 0.9 mL of Debris Removal Solution (Miltenyi, 130-109-398). Next, 4 mL of “CCS + Stop buffer” was slowly added on the top layer and the mixture was centrifuged at 3000*g* for 10 min. The supernatant was carefully discarded, ensuring not to disturb the cell pellet. The cell pellet was then resuspended in 10 mL of “CCS + Stop buffer” and centrifuged at 500*g* for 5 min. Finally, the cleaned cell pellet was resuspended in 0.2 mL of PBS [42].

### Droplet-based single-cell sequencing

Single cell sequencing was performed on brain cells from the cerebral cortex, striatum, and thalamus. cDNA libraries were prepared from single-cell suspensions using the 10 × Genomics 3′ V3 protocol. The single-cell suspensions were loaded onto a Chromium Single-Cell Controller Instrument (10 × Genomics) to generate single-cell gel beads in emulsions (GEMs). Approximately 15,000 cells were added to each channel to capture 10,000 cells per library. The GEMs underwent GEM-RT incubation, fragmentation, adaptor ligation, and sample index PCR. The resulting library was then sequenced on an Illumina NovaSeq.

### Quality control and cell type determination

The Cell Ranger Toolkit V.3.1.0, provided by 10x Genomics, was used to align reads and generate the gene-cell unique molecular identifier (UMI) matrix against the reference genome GRCm38, which was downloaded from the 10x Genomics official website. High-quality cells for downstream analysis were determined by retaining cells with gene and UMI numbers within the mean ± 2 times standard deviation range, and mitochondrial gene ratios below 30%. To reduce the dimensionality of the scRNA-Seq dataset, principal component analysis (PCA) was performed on an integrated data matrix. The top 50 principal components were used for downstream analysis, as determined by the Elbowplot function of Seurat. The main cell clusters were identified using the FindClusters function offered by Seurat, with the default resolution set as 0.4. Based on the differentially expressed genes (DEGs) for each cluster and conventional markers described in a previous study, the 34 cell clusters were grouped into 10 main cell types. Additionally, 4 immune-related cell types and neurons were subset and further clustered into subclusters to detect heterogeneity.

### Pseudotime trajectory analysis

Monocle2 package was used perform machine learning based on the expression patterns of key genes to simulate the dynamics of the temporal developmental process. Firstly, we select the genes that have a large degree of variation in gene expression between cells, perform spatial dimensionality reduction based on their expression profiles, and then construct a minimum spanning tree (MST), through which we find the longest paths that represent the differentiation trajectories of cells with similar transcriptional profiles [43].

### Cell–cell interaction

For scRNA-seq data, we used CellChat to identify the ligand-receptor interaction and cell–cell communication, CellChat analysis was performed using the R CellChat package (https://github.com/sqjin/CellChat) [44], Significant ligand-receptor pairs between cell types were defined as those with a P-value < 0.05.

### Flow cytometry

In flow cytometry analyses, Fc receptors were blocked for 5 min on ice using an anti-CD16/CD32 (BioLegend, 156603). To determine cell viability, Zombie NIRTM Fixable Viability Kit was used following the manufacturer’s instructions (BioLegend, 423105). Then the brain single cells were stained with the following antibodies in staining buffer (total volume 50 μL): FITC-conjugated CD3 (BD Bioscience, 555274), PE-conjugated CD4 (BD Bioscience, 553730), APC-conjugated CD8a (BD Bioscience, 553035), Percp5.5-conjugated CD45 (Thermo, 45-0451-80), PE-conjugated CD11b (BioLegend, 101207), APC-conjugated Ly6c (BioLegend, 128015), and FITC-conjugated Cd49b (BioLegend, 128015), Cx3cr1 (BioLegend, 149007). Or the cells were fixed and permeabilized using the BD Cytofix/Cytoperm™ Fixation/Permeabilization Kit (554714). Then, incubated with NeuN (Proteintech, 66836-1-ig) or Baiap2 (Abclonal, A5337) for 30 min at 4 °C, they were washed twice with staining buffer and incubated with Alexa Fluor 555-conjugated secondary antibody (Invitrogen, USA) for 30 min at 4 °C, followed by two washes with staining buffer. Subsequently, they were incubated with FITC- conjugated JEV-E antibody for 30 min at 4 °C, washed twice with staining buffer. Samples were resuspended in 200 μL of 1% BSA in staining buffer and analyzed using FlowJo (BD).

### Immunohistochemistry assay

Mice were deeply anesthetized by injection of ketamine hydrochloride and then perfused transcardially with 4% paraformaldehyde (PFA) in 0.1 M sodium phosphate buffer (1 × PBS). The brains were post-fixed for 1–3 days and then dehydrated and embedded in paraffin. Coronal and sagittal brain sections (5 μm) were obtained using a vibratome (Leica). To remove endogenous peroxidase, the slices were soaked in 3% H_2_O_2_ (Genetech, GT100534) for 10 min. Antigenic sites were exposed by steaming the slices in a microwave oven with citric acid buffer. Subsequently, the slices were incubated in 1 × PBS blocking solution containing 5% BSA for 1 h at room temperature. Finally, the slices were incubated overnight at 4 °C in the same solution containing primary antibodies at the following concentrations: 1:200 for JEV-E, 1:500 for Ly6c (BioLegend, 108401), 1:500 for CD4 (BioLegend, 100402), 1:500 for CD8a (BioLegend, 372902), and 1:500 for CD49b (BioLegend, 108901), 1:500 for Iba1 (Abcam, ab178846). The next morning, the sections were washed three times for 5 min in 1 × PBS. Then, they were incubated for 45 min at room temperature in HRP-labelled sheep anti-mouse/rabbit IgG (Genetech, GK500505A). After that, the sections were washed three more times for 5 min in 1 × PBS. The freshly prepared DAB working solution (Genetech, GK347010) was added to cover the whole tissue, and the color development was controlled under the microscope. The slices were then re-stained with hematoxylin for 2 min. Once dried, the slides were coverslipped and sealed. Finally, the slides were scanned using the Leica Apero CS2 slice scanning system.

### Treble-fluorescence immunohistochemical assay

Brain tissues were embedded in OCT and frozen using liquid nitrogen before being stored at − 80 °C for further processing. Sagittal slices (10 μm) were prepared using a freezing microtome (Leica). Treble-Fluorescence immunohistochemical staining was performed following the manufacturer’s instructions (RS0035, ImmunoWay, China). The mounted slices were dewaxed and repaired in a repair box. Tissue sections were then removed and rinsed 5–6 times with distilled water. Peroxidase enzyme closure buffer was added dropwise and incubated for 15 min at room temperature, followed by rinsing with PBS three times for 2 min each. The tissue was circled using an immunohistochemical pen, and the corresponding primary antibody was added dropwise until the tissue was completely covered. Incubation was done at room temperature or 37 °C for 1–2 h, or overnight in a wet box at 4 °C. Afterward, the tissue was rinsed with PBS three times for 2 min each. HRP polymeric anti-rabbit/mouse secondary antibody was added dropwise and incubated for 30 min at room temperature, followed by rinsing with PBS three times for 2 min each. Finally, the fluorescent dye reagent D-594 working solution (100 µl) was added, and after 10 min, the tissue was rinsed with PBS three times for 2 min each. The sections should be placed in the repair box and treated with the stripping solution. Then, they should be microwaved for 3 min on high and 15 min on medium–low. After cooling naturally, the sections should be rinsed with PBS for 2 min, repeating this step three times. Next, the previous steps should be repeated, but this time the intermediate fluorescent dye should be changed to Reagent D-488 and the second primary antibody should be incubated. Following that, the intermediate fluorochrome should be changed to Reagent D-647 and the third primary antibody should be incubated. Finally, a drop of Reagent (antifade mounting medium with DAPI) should be applied on the tissue section, the coverslip should be covered, and the section should touch the sealing solution to avoid air bubbles as much as possible. The images were acquired using a confocal microscope (N-STORM, Nikon, Japan).

### Fluorescence in situ hybridization

Hybridization probes were designed by Spatial-FISH (Shenzhen, China), selecting target regions with high hybridization efficiency and specificity from the RNA sequences to be detected. For sample hybridization, a reaction chamber was prepared on tissue sections of the samples to be detected. The PFA-fixed slides were dehydrated and denatured with 100% methanol, and the hybridization buffer was added to the reaction chamber and incubated at 37 °C overnight. For the ligation reaction, the hybridization buffer was discarded, and the reaction chamber was washed three times with PBST. Then, the ligase system was added to the reaction chamber and incubated at 25 °C for 3 h. After the ligation reaction, the ligase system was discarded, and the reaction chamber was washed several times with PBST. Next, the rolled-loop amplification system was added to the reaction chamber and incubated overnight at 30 °C. Following the rolled-loop amplification, the amplification system was discarded, and the reaction chamber was washed several times with PBST. Finally, the fluorescence probe hybridization system was added to the reaction chamber for hybridization. In situ color development, gradient alcohol dehydration, and sealing were performed. Microscopic imaging was then conducted to decipher the RNA spatial location information in the study region of the sample to be tested.

### RNA extraction and real-time reverse transcription (RT)-PCR

Total RNA was extracted from treated cells with TRIzol reagent (Invitrogen, USA) according to the manufacturer’s instructions, and the first-strand cDNA was synthesized using the ABScript II cDNA First-Strand Synthesis Kit (ABclonal, China). Relative quantitative PCR (qPCR) was performed using 2X University SYBR Green Fast qPCR Mix (ABclonal, China). Primers JEV-C-F/R were used for relative analysis of viral replication. JEV-C-F: GGCTTTTATCACGTTCTTCAAGTTT; JEV-C-R: TGCTTTCCATCGGCCTAAAA. The absolute qPCR was performed with the primers JEV-E-F/R and probe. JEV-E-F: TGGTTTCATGACCTCGCTCTC; JEV-E-R: CCATGAGGAGTTCTCTGTTTCT; Probe: CCTGGACGCCCCCTTCGAGCACAGCGT. The standard curve was performed with tenfold serial diluted plasmid containing JEV E gene.

### Western blotting

Cells were harvested and incubated in lysis buffer (Beyotime Biotechnology, Shanghai, China) containing protease inhibitor on ice for more than 30 min, a Cell lysate was centrifuged for 15 min at 13,000 rpm at 4 °C, and the supernatant was measured with a bicinchoninic acid protein assay kit (BCA, Thermo Scientific, USA). The proteins were separated by SDS-PAGE, transferred to nitrocellulose membrane. The membrane was blocked by 5% BSA for 1 h at room temperature and incubated with primary antibody at 4 °C overnight. The next day, the membrane was washed three times with 1xTBST and incubated with horseradish peroxidase-conjugated secondary antibodies for 1 h at room temperature and visualized with an enhanced chemiluminescence system (Tanon, China).

### Statistical analysis

All statistical analyses and graph generation were performed in R (version 3.6.0) and GraphPad Prism (version 8.0). The statistical analysis of differences between two groups was performed using two-tailed Student’s t-test. For all statistical significance indications in this manuscript, ****, p < 0.0001; ***, p < 0.001; **, p < 0.01; *, p < 0.05 and ns, no significance.

## Results

### Distributions of JEV in brain regions of infected mouse

To establish the JEV-infected mouse model and understand the viral tropism in brain regions, 5–6-week-old C57BL/6 mice were intraperitoneally injected with 10^5^ PFU JEV or equal volume of Dulbecco’s modified Eagle’s medium (DMEM). A significant reduction of body weight and obvious clinical symptoms were observed in mice after JEV infection (Additional file 1: Fig. S1A). Interestingly, the JEV-infected mice developed different symptoms during our observation. At 7 days post infection (dpi), some mice only showed mild symptoms, such as ruffled fur or hindlimb weakness, while some mice appeared severe symptoms like quiver and paralysis (Additional file 1: Fig. S1B, C). The brain tissues of mice exhibiting different symptoms were isolated at 7 dpi. The viral loads and the infected regions in the brain were determined by plaque assay and immunohistochemistry (IHC) assay respectively. An elevation on viral titers was observed in the brains of mice displaying severe symptoms, as compared to those in mice with mild symptoms (Additional file 1: Fig. S1D). In addition, the IHC analysis with the sagittal and coronal brain slice revealed that JEV was predominantly present in the cortex, striatum, and thalamus. As symptoms worsened, an increase in the abundance of viral protein in the brain was detected (Additional file 1: Fig. S1E, F).

### Single-cell transcriptional profiling of JEV-infected mouse brain

**Fig. 1:**
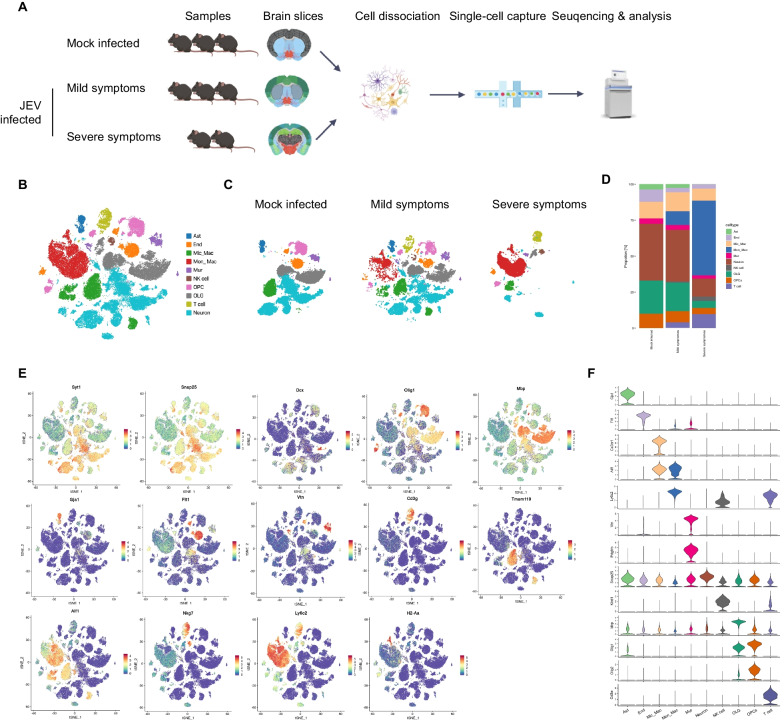
Changes of cell types upon JEV infection in mouse brain revealed by scRNA-Seq. **A** Schematic diagram showing the isolation of single cells from cerebral cortex, striatum and thalamus of mock- and JEV-infected mice for scRNA-seq. **B** Overview of the cell types in the integrated single-cell transcriptomes of 88,000 cells derived from mock- and JEV-infected mouse brain. **C** tSNE plots of cell types from different symptoms. **D** Proportion of different cell types in mice with different symptoms. **E** tSNE plot colored for gene expression in different cell types. **F** The marker genes used to define each cell type are illustrated in the violin plot

To captures the transcriptional landscape of various cell types in the brain tissue of mice developing different symptoms following JEV infection, the scRNA-seq (10 × Genomic) technology was employed (Fig. 1A). 5–6-week-old C57BL/6 mice were intraperitoneally with JEV (JEV-infected group, n = 5) or DMEM (mock-infected group, n = 3), and the body weight of mice was recorded daily for 15 consecutive days (Additional file 2: Fig. S2A). At 7 dpi, 3 mice presented mild symptoms while 2 mice displayed severe symptoms. The single cells were isolated from the cortex, striatum and thalamus areas of the brain in JEV-infected mice with different symptoms, as well as mock-infected mice, on 8 corona slices (Additional file 2: Fig. S2B). Single cell suspension was then visualized under a microscope, and the cell viability was examined. The results showed that we successfully obtained a sufficient number of single cells with over 90% viability (Additional file 2: Fig. S2C, D), and copies of JEV E gene in the single cell was measured (Additional file 2: Fig. S2E).

After performing scRNA-seq, we obtained a total of 88,000 transcriptomes from single cells in the brain tissues of 3 mock-infected mice and 5 JEV-infected mice exhibiting mild and severe symptoms. By analyzing the top differentially expressed genes (DEGs) (Additional file 3: Fig. S3B), we identified 34 clusters (Additional file 3: Fig. S3A–C) and 10 major cell types (Fig. 1B–D). These included astrocytes (Ast) (Gia1+), endothelial cells (End) (Flt1 + Cldn5 +), microglia & macrophages (Mic_Mac) (Tmem119 + Cx3cr1 + Aif1 +), monocytes & macrophages (Mon_Mac) (CD14 + Ly6c2 +), mural cells (Mur) (Vtn + Pdgfrb +), neurons (Snap25 + Syt1 + Tubb3 +), natural killer (NK) cells (Nkg7 + Ccl5 +), oligodendrocytes (OLG) (Plp1 + Mbp +), oligodendrocyte precursor cells (OPCs) (Olig1 + Olig2 +), and T cells (CD3g + CD3g + CD3e +) (Fig. 1C–F). Our results revealed that as the symptoms worsened following JEV infection, there was a progressive increase in immune-related cells, whereas the population of neurons and glial cells gradually declined in mouse brain (Additional file 3: Fig. S3D, E). In the group with mild symptoms, Mon_Mac, T and NK cells started to emerge, and a new cluster of mural cells and endothelial cells appeared. Additionally, a new cluster of OPCs, OLG and Mic_Mac emerged. In brains of mice with severe symptoms, Mon_Mac accounted for more than half of the cells, and a notable reduction in the number of neurons and glial cells was observed (Fig. 1C, D).

**Fig. 2:**
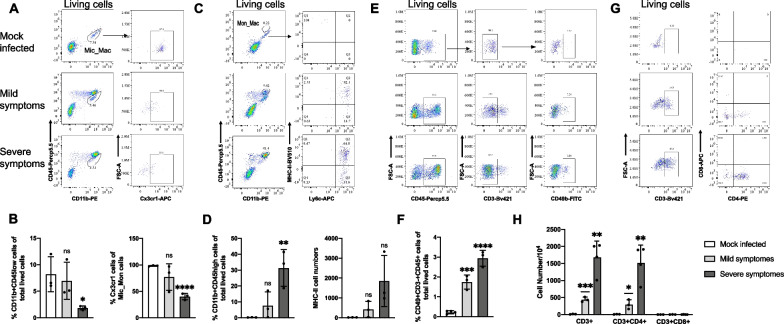
Flow cytometry analysis of immune cells in brains of mice with different symptoms upon JEV infection. **A**, **C**, **E**, **G** Flow cytometry gating strategy of Mic_Mac (**A**), Mon_Mac (**C**), NK cells (**E**) and T cells (**G**). **B** Percentage of CD11b + CD45low cells in total living cells and percentage of Cx3cr1 + cells in Mic_Mac cells. **D** Percentage of CD11b + CD45high cells in total living cells and the number of cells that secrete MHC-II. **F** Percentage of CD49+ CD3-CD45+ cells in total living cells. **H** Cell numbers of CD3+, CD4+ CD3+, and CD8a + CD3+ cells. Data from 3 independent experiments were presented as mean ± SEM. The statistics were analyzed using two-tailed Student’s t-test. *P < 0.05, **P < 0.01, ***P < 0.001, ****P < 0.0001. ns: nonsignificant

To validate the scRNA-seq results, flow cytometry was performed to detect the presence of Mic_Mac, Mon_Mac, T cells, and NK cells. As the severity of symptoms increased, the proportion of Mic_Mac (CD11b + CD45low) among total viable cells was decreased. Additionally, the ratio of Cx3cr1-positive cells, which are known as resting microglia cells, was reduced among Mic_Mac, indicating that most Mic_Mac were activated (Fig. 2A, B). In consistent with the scRNA-seq results, Mon_Mac (CD11b + CD45high) showed a dramatical increase, accompanied by a notable rise in the number of cells expressing the major histocompatibility complex class II (MHC-II), particularly in mice exhibiting severe symptoms (Fig. 2C, D). Similarly, the proportion of NK cells, identified using the CD45+ CD3−CD49+ approach, also showed a marked elevation (Fig. 2E, F). Furthermore, we identified T cells by labeling CD4+ T cells as CD45+ CD3+ CD4+ and CD8+ T cells as CD45+ CD3+ CD8a+. We observed a significant increase in total number of T cells after JEV infection. Notably, the CD4+ T cells were found to be more enriched than CD8+ T cells (Fig. 2F, G) [45, 46]. To further confirm these findings, the IHC assay was performed, and consistent changes on Mon_Mac, Mic_Mac, T cells and NK cells were observed (Additional file 4: Fig. S4). Taken together, these results suggest that after JEV infection, most Mic_Mac in the brain were activated, and the immune cells such as Mon_Mac, NK and T cells distributed near the blood vessels were significantly increased.

### Dynamic composition and functional changes of brain cells during JEV infection

**Fig. 3:**
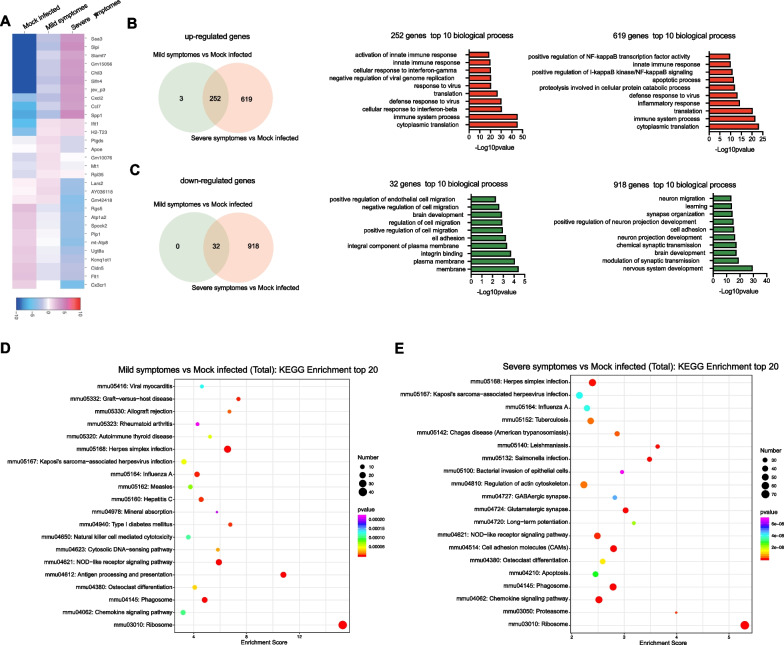
Changes of gene expression in JEV-infected mouse brain. **A** The top 5 significantly altered genes (rows) in each group. **B**, **C** Venn diagram and GO analysis of overlapping up- (**B**) and down-regulated (**C**) genes across different symptoms. **D**, **E** Analysis of the top 20 KEGG pathways

The scRNA-seq results showed significant alterations in cell composition and gene expression among different groups. As symptoms worsen, an increasing amount of differentially expressed genes was observed (Fig. 3A). The Venn diagram revealed that the intersection of the upregulated genes between mice with mild symptoms compared to mock-infected mice and mice with severe symptoms compared to mock-infected mice are associated with cytoplasmic translation and immune system process, while the down-regulated genes are related to the plasma membrane (Fig. 3B). In addition, as the symptoms worsened, the number of newly differentiated genes gradually increased, among which the upregulated genes were found to be related to autophagy and NF-κB pathways, while the downregulated genes are associated with nervous system development (Fig. 3B, C). Furthermore, compared with the mock-infected group, the top 20 KEGG enrichments were related to antigen processing and presentation in mild symptoms (Fig. 3D). While in severe symptoms, differentially regulated genes were predominantly enriched in cell adhesion molecules, phagosomes, and the chemokine signaling pathway (Fig. 3E).

In order to understand the responses of different cells in mouse brain to viral infection, further analysis was conducted to compare the differentially regulated genes between the JEV-infected and mock-infected groups. It was observed that common upregulated genes were shared across different cell types (Additional file 5: Fig. S5), indicating a general response to the virus. These upregulated genes are involved in various processes such as response to interferon, cytokine production, cytosolic ribosome, and negative regulation of the immune system. In contrast, the downregulated genes showed cell-type specific changes. For example, the function of downregulated genes in endothelial cells is associated with vasculature development. Similarly, downregulated genes in mural cells regulate cell adhesion, while downregulated genes in neurons are involved in myelination. Additionally, downregulated genes in oligodendrocytes and oligodendrocyte precursor cells are linked to axon.

### Cell–cell communications in JEV-infected mouse brain

**Fig. 4:**
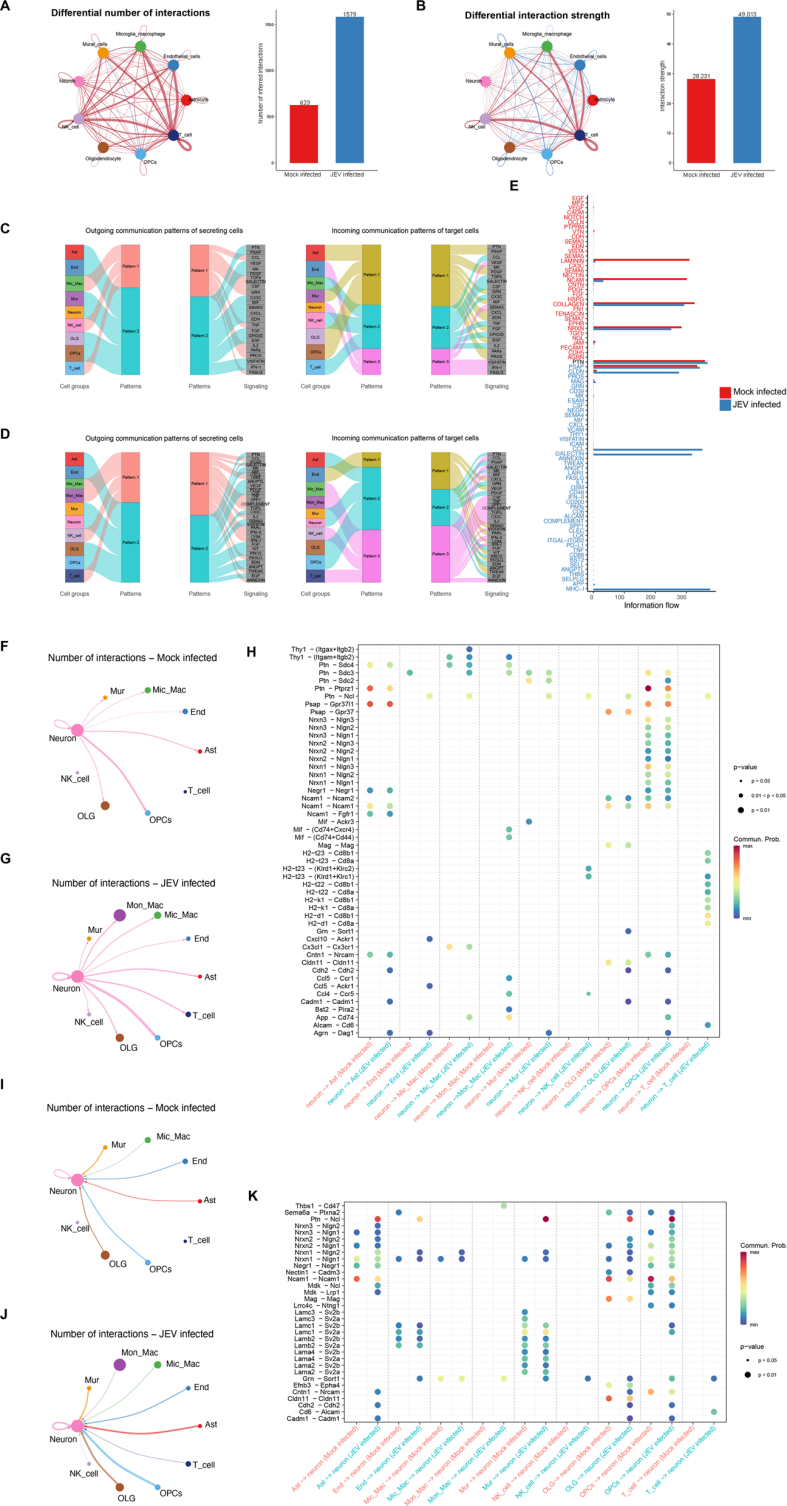
Cell–cell communications in JEV-infected mouse brain. **A**, **B** Circle plot showing the number of interaction (**A**) and interaction strength (**B**) of different cells in brain. The line thickness represents the number and the strength of signaling. The red lines represent JEV-infected mouse brain and blue lines represent the mock-infected mouse brain. Histogram shows interactions number and strength interaction. **C**, **D** River plot showing outgoing communication pattern of secreting cells and incoming communication pattern of target cells in mock-infected group (**C**) and JEV-infected group (**D**). **E** Information flow of each signaling pathway from cell–cell interaction analysis. The receptor-ligand pathways with blue text are significantly enriched in JEV-infected brain cells, and pathways with red text are significantly enriched in mock-infected mouse brain cells. **F**, **G** Number of neuronal cells communicating with other cells as ligands in mock-infected group (**F**) and JEV-infected group (**G**). **H**, **K** Bubble plots show the variable ligand-receptor pairs between neuronal cells and other cells, when the neurons are ligands (**H**) or receptors (**F**). **I**, **J** Number of neuronal cells communicating with other cells as receptors in mock-infected group (**I**) and JEV-infected group (**J**)

To further understand the communications between different types of cells in the brain tissues of mice during JEV infection, Ligand-receptor analysis and visualization were performed using CellChat version 1.6.0. A higher number of inferred interactions and the interaction strength was found in JEV-infected group compared to that in the mock-infected group (Fig. 4A, B). Among the interactions, T cells showed the highest frequency and strength of interactions with other cells, particularly with endothelial cells, Mic_Mac, and NK cells (Fig. 4A, B). Then, we conducted a communication pattern analysis in both mock- (Fig. 4C) and JEV-infected groups (Fig. 4D), revealing the two patterns in outgoing secreting cells and three patterns in incoming target cells. Pattern 1 was identified in outgoing Mic_Mac, NK and T cells signaling, involving multiple pathways, such as chemokine (C-C motif) ligand (CCL), GALECTIN, colony stimulating factor (CSF), tumor necrosis factor (TNF), interleukin 2 (IL2) and FASLG. In JEV-infected group, new pathways such as CXCL, secreted phosphoprotein 1 (SPP1), protease activated receptors (PARS) and oncostatin-M (OSM) emerged, contributing to inflammation, immune responses and cell proliferation or differentiation. Outgoing astrocyte, endothelial cells, mural, neuron, oligodendrocytes, and oligodendrocyte precursor cells signaling was identified in pattern 2. Both the mock- and JEV-infected groups were enriched in pleiotrophin (PTN), prosaposin (PSAP), Midkine (MK), and CX3C, which are involved in information transmission and neuronal repair functions. In addition, there is a significant difference in the incoming communication patterns between mock- and JEV-infected groups. In mock-infected group, both endothelial cells and neurons showed consistent pattern. However, in JEV-infected groups, endothelial cells presented a separate pattern. This separate pattern represents pathways such as Protease Activated Receptors (PARs), OSM, KIT, angiopoietin (ANGPT), and tumor necrosis factor-like weak inducer of apoptosis (TWEAK), which are associated with inflammation, vascular regeneration, and regulate cell growth and development. Neuron, astrocyte, mural, oligodendrocytes and oligodendrocyte precursor cells were identified as part of a different pattern that represents the endothelin (EDN) pathway, whereas Mic_Mac, Mon_Mac, NK, and T cells were found to be associated with pattern3, which is linked to the TGFb and IFN-II pathways.

In addition, we investigated the signaling pathways potentially involved in cell communications in mock- and JEV-infected groups. We found that laminin and neural cell adhesion molecule (NCAM) signaling, which play a crucial role in regulating cell growth and differentiation, were particularly activated in mock-infected group (Fig. 4E). On the other hand, the JEV-infected group showed upregulation of claudin (CLDN, a vital component of epithelial cell tight junctions), CCL (related to inflammation), Galectin (involved in regulating key biological processes including cell growth, differentiation, apoptosis, and immune responses), and MHC-I (primarily responsible for mediating the cytotoxic effect of T cells) signaling pathways (Fig. 4E). These results suggest that JEV infections not only result in neuronal damage and disruption of the cellular barrier, but also trigger the body’s immune response and secretion of various cytokines to combat the viral infections.

As neurons have been identified as the primary target cells of JEV in the CNS, studying their interactions with other cells is crucial for understanding JEV pathogenesis. Interestingly, we observed that when neurons act as ligands, neuron presented the most frequent interactions with OPCs, both in mock- (Fig. 4F) and JEV-infected groups (Fig. 4G), the Ptn secreted by neurons primarily interacts with Sdc1/2/3 and Ptprz (Protein Tyrosine Phosphatase, Receptor Type Z) on the surface of OPCs, thereby regulating neuronal growth and development (Fig. 4H). Additionally, H2-t23, H2-t22, H2-k1, and H2-d1 secreted by neurons could interact with Cd8a and Cd8b1 on the surface of T cells. Moreover, the neurons were also predicted to communicate with other immune cells. For instance, H2-t23 from neurons was shown to interact with Kldr1 + Klrc2 and Kldr1 + Klrc1 on the surface of NK cells, while Mif and App interact with Cd74 on Mic_Mac and Mon_Mac cells (Fig. 4H). When the neuron acts as a receptor, OLG and OPCs presented the most frequent interactions with neurons, both in mock- (Fig. 4I) and JEV-infected groups (Fig. 4J). Upon JEV-infection, the expression of Ncl, which receives Ptn signal from glial cells and mural cells, was upregulated in neurons, while Ncam1 and Cldn11, which are involved in cell adhesion and tight junction, were found to be downregulated in neurons (Fig. 4K). The findings indicate that JEV infection in neurons triggers the secretion of certain molecules by glial cells, which in turn promote neuronal growth and development while decreasing neuronal adhesion. Additionally, neurons engage in interactions with immune-related cells to facilitate the clearance of the virus.

### Heterogeneous subpopulations of immune cells in JEV-infected mouse brain

**Fig. 5:**
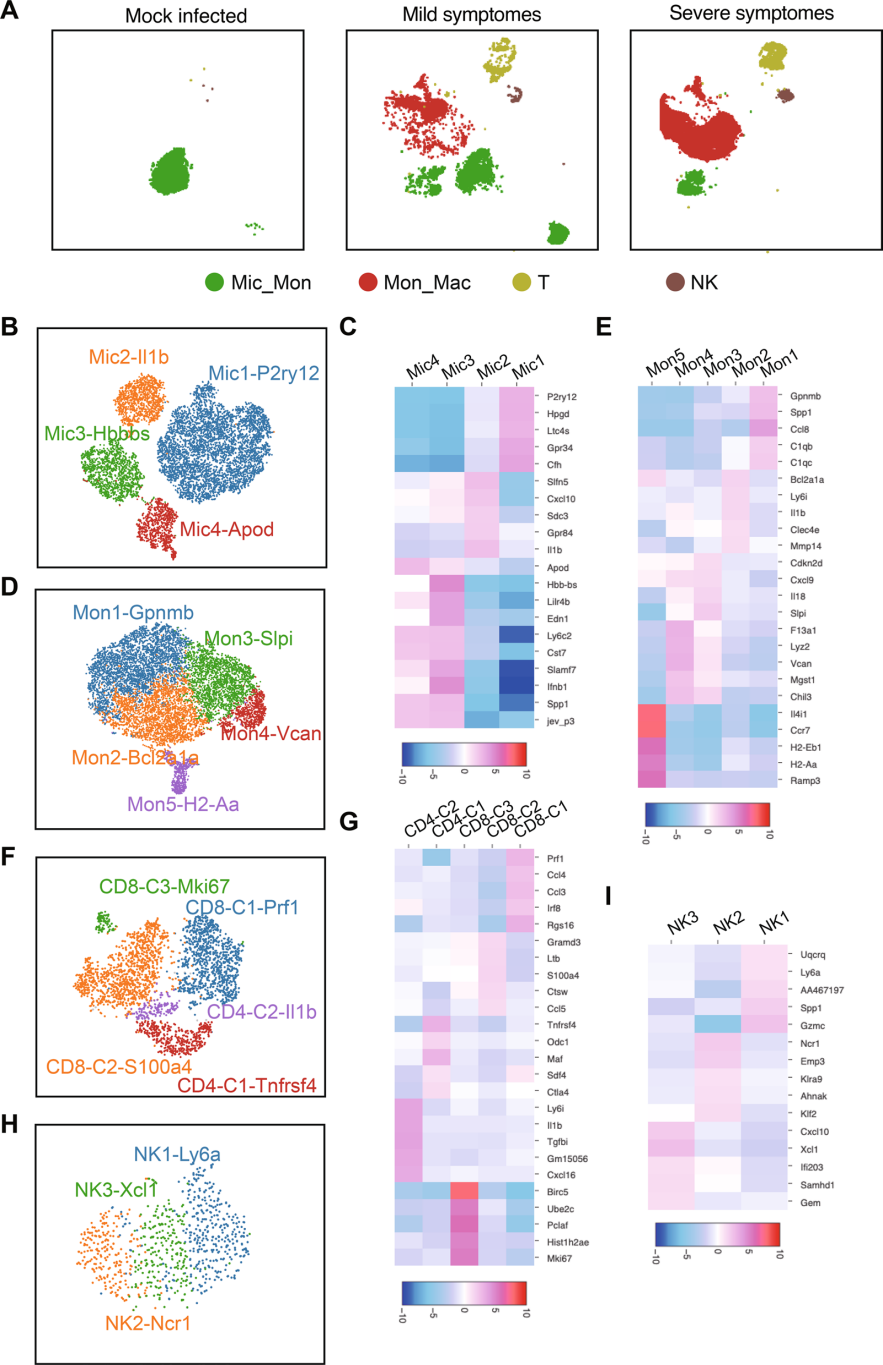
Subclusters of immune cells and the potential developmental trajectory of cell subsets. **A** tSNE visualization of immune related cell populations in different group. **B**, **D**, **F**, **H** Overview of the subclusters of Mic_Mon (**B**), Mon_Mic (**D**), T (**F**), and NK (**H**) cells. The subclusters were named based on the cluster-specific gene expression patterns. **C**, **E**, **G**, **I** Heatmap of differentially regulated genes among subtypes of Mic_Mon (**C**), Mon_Mic (**E**), T (**G**), and NK (**I**) cells

Considering the notable variations in the composition of immune related cells in mouse brains of different groups (Fig. 5A), we conducted the subclass analysis on these cells to understand the involvement of different subpopulations of immune cells in JEV infection. Mic_Mac cells, which are major immune cells in the brain, have been reported to play a crucial role in the progression of JEV infection. In this study, we identified six clusters of Mic_Mac cells (Additional file 6: Fig. S6A). Clusters 2 and 4 were primarily found in the brains of mock infected group, while cluster 1 was identified in both mock-infected and mildly symptomatic groups. Cluster 5 was predominantly observed in the brains of mice with mild symptoms, whereas clusters 3 and 6 were mostly enriched in those with severe symptoms (Additional file 6: Fig. S6B, C). By comparing the gene expression from different clusters, we found that clusters 1, 2, and 4 shared similar gene profiles related to anti-inflammatory M2 type macrophages. Based on the highly expressed genes in these cells, we designated these cells as Mic1-p2ry12 (Fig. 5B). On the other hand, clusters 3, 5, and 6 displayed a more M1 type-dominant gene signature, such as the high expression of Ccl2, Isg15 and Ifi211 (Additional file 6: Fig. S6C). These clusters of cells were named Mic2-Il1b, Mic3-Hbb-bs and Mic4-Apod, respectively, based on their differentially expressed genes (Fig. 5B, C). Conversely, clusters 1, 2, 4, and 5 exhibited high expression levels of Tmem119 and Cx3cr1, suggesting a resting state of microglia. Interestingly, clusters 3 and 6 showed increased expression of Aif1 (iba1) after JEV infection, indicating a transformation of microglia from a resting state to an active state (Additional file 6: Fig. S6C).

To investigate the evolutionary trajectory of Mic_Mac cells, we conducted a pseudo-time analysis using Monocle 2. In this analysis, we observed a similar phenomenon where Mic_Mac cells from mock-infected mice were predominantly found at the beginning of the trajectory (Additional file 6: Fig. S6D, E). However, the trajectory of Mic_Mac cells from mice with mild symptoms deviated from that of mock-infected mice, while the opposite end of the trajectory was comprised of Mic_Mac cells from mice with severe symptoms. Furthermore, we also analyzed the cells at the branch point, both before and after differentiation. We generated a heatmap of the differentially enriched genes, allowing the identification of four modules. Afterwards, we performed individual GO enrichment and KEGG analysis on these modules (Additional file 6: Fig. S6F). The analysis of module 1 and 2 revealed that the developmental track of Mic_Mac cells in cell fate 1 primarily involved inflammation responses, cytokine activity, and viral protein interaction with cytokine and cytokine receptor. In cell fate 2, it was mainly associated with the chemokine-mediated signaling pathway.

Mon_Mac cells, as the most diverse cell population after virus infection, exhibited notable heterogeneity with distinct clusters (Additional file 7: Fig. S7A, B). These subclusters were named based on their gene expression patterns (Fig. 5D). In the Mon1-Gpnmb cluster, the complement system genes C1qb and C1qc were significantly up-regulated (Fig. 5E), indicating their involvement in apoptosis. The Mon2-Bcl2a1a cluster showed high expression of Il1b, Bcl2a1a, and Ly6i, which are associated with the NF-κB signaling pathway. In addition, the Mon3-Slpi and Mon4-Vcan clusters were found to be associated with inflammation, while the Mon5-H2-Aa cluster exhibited expression of H2-Eb1, H2-Aa, and Ccr7, indicating its involvement in antigen presentation (Additional file 7: Fig. S7C). Furthermore, single-cell trajectory analysis identified clusters 4 and 5 as the earliest cells to emerge during disease development (Additional file 7: Fig. S7D). Functional enrichment analysis suggested that these clusters were associated with inflammatory response and antigen processing and presentation. Subsequently, cluster 3 emerged, characterized by genes involved in negative regulation of viral replication. On the other end of the trajectory, clusters 1 and 2 were enriched with genes participating in the mTOR signaling pathway and NK cell-mediated cytotoxicity (Additional file 7: Fig. S7D–F).

A total of 3320 T cells were detected in all samples and classified into 9 clusters using t-SNE. The majority of cells were derived from individuals with mild and severe symptoms (Additional file 8: Fig. S8A, B). Based on the expression of marker genes of specific T cell subtypes, 2 clusters were identified as CD4+ T cells (20.66%), namely CD4-C1-Tnfrsf4 and CD4-C2-Il1b. 7 clusters were identified as CD8+ T cells (79.34%), including the CD8-C1-Prf1 cluster, associated with cytotoxic T cells, the CD8-C2-S100a4 cluster, representing effector memory T cells, and the CD8-C3-Mki67 cluster, known as proliferative T cells (Fig. 5F and G).We further investigated the dynamic immune states of T cells using Monocle. The cells were distributed on a trajectory chart consisting of three branches (Additional file 8: Fig. S8D, E). We subsequently analyzed the expression patterns of all identified genes during the progression of T cell exhaustion. As a result, we identified four modules by generating a heatmap of the differentially enriched genes. The CD4-C2-Il1b and CD8-C2-S100a4 cells, belonging to module 3 were observed at the starting point of the pseudotime (Additional file 8: Fig. S8E). GO analysis indicated that their function is mainly associated with defense response to viruses and response to interferon-beta (Additional file 8: Fig. S8E). Cell fate 1 was shown at the upper left of the branch point, which is enriched with CD8-C1-Prf1 and CD4-C1-Tnfrsf4. The genes enriched in this module are associated with viral protein interaction with cytokines and cytokine receptors. The other end of the trajectory is populated with CD8-C2-S100a4 cells, which are mainly related to natural killer cell-mediated cytotoxicity and adaptive immune response (Additional file 8: Fig. S8F).

The number of NK cells was relatively low, with a count of 842 cells, and the majority of these cells were derived from JEV-infected mice (Additional file 9: Fig. S9A–C). In this study, NK cells were divided into three subpopulations based on their expression of Ly6a, Ncr1, and Xcl1, instead of the traditional classification into CD56bright and CD56dim subpopulations (Fig. 5H, I). Pseudotime analysis revealed that NK cells from each of the 3 clusters were assigned to the 3 different developmental trajectories (Additional file 9: Fig. S9D). It was found that Ncr1 + NK cells were in the early stage of the developmental trajectory (Additional file 9: Fig. S9E). Along cell fate 1, NK cells develop with a focus on antiviral activity and cytotoxicity. Along cell fate 2, they play a role in immune regulatory functions by regulating cytokine interactions and T cell proliferation (Additional file 9: Fig. S9F).

### The cell types susceptible to JEV in the CNS

**Fig. 6:**
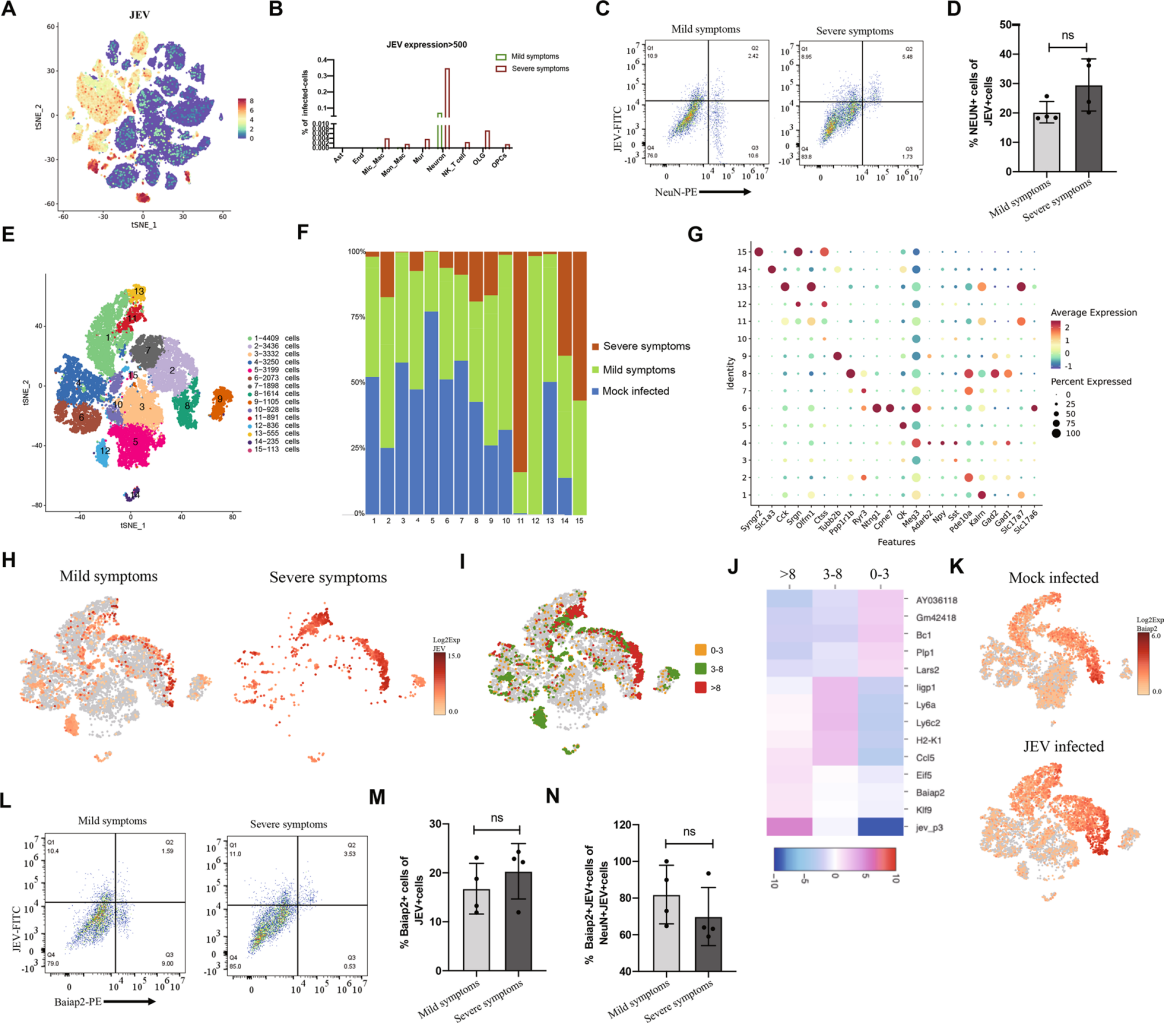
Baiap2 positive neurons exhibit high susceptibility to JEV. **A** Distribution of JEV genome in different cells. **B** Percentage of JEV-infected cells (the count of JEV gene > 500) in each cell type. **C** Flow cytometry for detection of JEV E protein and NeuN in brain cells of mice infected with JEV. **D** The histograms summarizing the indicated cell populations of JEV-infected neurons (n = 4). **E** tSNE visualization of clustering revealed 15 distinct neuronal populations. **F** Histogram showing the proportions of cells in each group and each subcluster. **G** Dot plot showing the expression of representative markers that define the cluster in neurons. **H** tSNE visualization of JEV gene expression in neuron cells and heatmap of JEV transcript positive cells separated by different symptoms. **I** Heatmap showing the neurons with different abundance of JEV genomic RNA. **J** The top 5 genes in brain cells infected with different amount of JEV. Different color dots represent viral gene expression, yellow dot: low expression (Log2 Exp JEV less than 3); green dot: medium expression (Log2 Exp JEV between 3 and 8); red dot: high expression (Log2 Exp JEV more than 8). **K** Heatmap of Baiap2 expression in neurons in mouse brain. **L** Flow cytometry analysis of JEV and Baiap2 positive cells in brains of mice with different symptoms after JEV infection. **M** The histograms summarizing the ratios of indicated cells infected with JEV in the Baiap2 positive neurons (n = 4). **N** The histograms summarizing the ratio of Baiap2 and JEV positive cells in NeuN and JEV positive cells. Data from 3 independent experiments were presented as mean ± SEM. The statistics were analyzed using two-tailed Student’s t-test. ns: nonsignificant

Although neurons have long been recognized as the primary target cells of JEV in the CNS, the specific subtype of neurons that JEV prefers and the susceptibility of other cell types to JEV remain uncertain. Our analysis of the scRNA-seq data revealed that neurons exhibit the highest level of infection (Fig. 6A), which aligns with previous findings. However, we also observed that other cells in the brain, such as glia cells, mural cells, and infiltrating cells, can also be infected by JEV (Fig. 6B). Flow cytometry analysis verified these findings, indicating a higher proportion of infected neuronal cells as the symptoms become more severe (Fig. 6C, D).

Given the high prevalence of infection in neurons, we further investigated the susceptibility of different subtypes of neurons to JEV. We performed subcluster on a total of 27,874 neurons, resulting in 15 subsets (Fig. 6E). After viral infection, subclusters 11, 12, and 15 emerged, while the remaining 12 clusters were present both before and after infection (Fig. 6F). Notably, each cluster exhibited specifical expression of neuron-related markers (Fig. 6G). Furthermore, we analyzed the expression of viral genes in different neuronal subtypes and found that it was not confined to a particular subgroup. Instead it was expressed in multiple subgroups, showing noticeable differences in expression levels (Fig. 6H). As the classification of neurons into five types, namely glutamatergic, aminobutyric acid, dopaminergic, serotoninergic, and cholinergic neurons, is well-established based on their secretion of neurotransmitters, we initially analyzed the susceptibility of these neuronal types to JEV according to the scRNA-seq results. Our findings revealed that both glutamatergic and aminobutyric acid neurons were susceptible to JEV infection (Additional file 10: Fig. S10). However, dopaminergic and serotoninergic neurons were not detected in the scRNA-seq results. To further determine the subtype of neuron that JEV prefers most, we classified neurons into three groups based on the level of viral gene expression: low expression (Log2 Exp JEV less than 3), medium expression (Log2 Exp JEV between 3 and 8), and high expression (Log2 Exp JEV more than 8) (Fig. 6I). By analyzing differentially expressed genes, we found a strong correlation between the level of Baiap2 expression and JEV infection (Fig. 6J, K). The presence of expression of Baiap2 and viral genes in the brain tissues of both JEV-infected and mock-infected mice indicates that the increased expression of Baiap2 was not attributed to JEV infection. By conducting flow cytometry, we found as symptoms worsen, the proportion of Baiap2-positive (Baiap2+) cells in all JEV positive cells increased (Fig. 6L, M). Furthermore, compared with severe symptoms, mild symptoms have a higher proportion of Baiap2 and JEV positive cells in all NeuN and JEV positive cells (Fig. 6N), which supports that the tropism of JEV to Baiap2-positive cells is not caused by the upregulation of Baiap2 upon JEV infection.

**Fig. 7:**
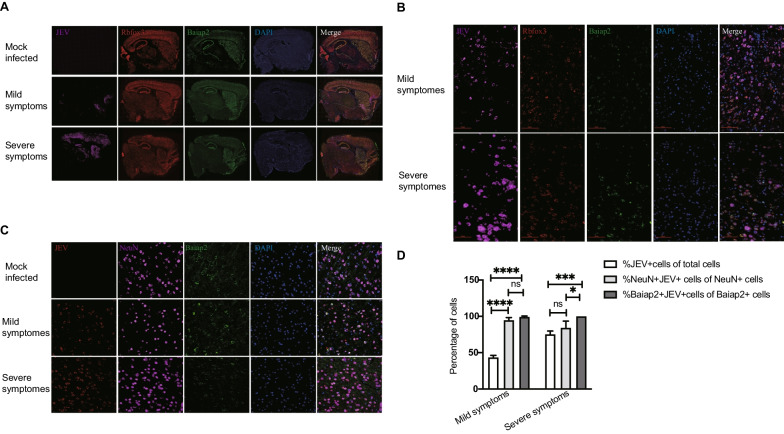
The expression of JEV genes is positively correlated with the expression of Baiap2 in neurons. **A** Detection of JEV, Rbfox3 and Baiap2 mRNA in brain coronal sections of mice with different symptoms by fluorescence in situ hybridization. **B** Enlarged view of area in **A**. (slice = 10 μm, Scale bars = 500 μm). (**C**) Detection of the expression of JEV E protein, Baiap2 and NeuN in mouse brain. (slice = 5 μm, Scale bars = 200 μm). **D** Statistical analysis of percentage of Baiap2 and JEV positive cells in NeuN and JEV positive cells in brain sections. Data from 3 slices were presented as mean ± SEM. The statistics were analyzed using two-tailed Student’s t-test. *P < 0.05, ***P < 0.001, ****P < 0.0001. ns: nonsignificant

To further verify the correlation between Baiap2 expression and JEV infection, we performed the fluorescence in situ hybridization (FISH) and IHC assays. The results of FISH assay with brain tissue sections showed that more viral RNA was detected in Baiap2 mRNA positive neurons (Fig. 7A, B). Similarly, the IHC assay revealed that more JEV E protein was observed in Baiap2 + neurons, and the proportion of JEV E positive cells in Baiap2 + neurons increased with worsening of symptoms (Fig. 7C, D). These results suggest that JEV predominantly infected Baiap2 + neurons (Fig. 7C, D).

## Discussion

**Fig. 8:**
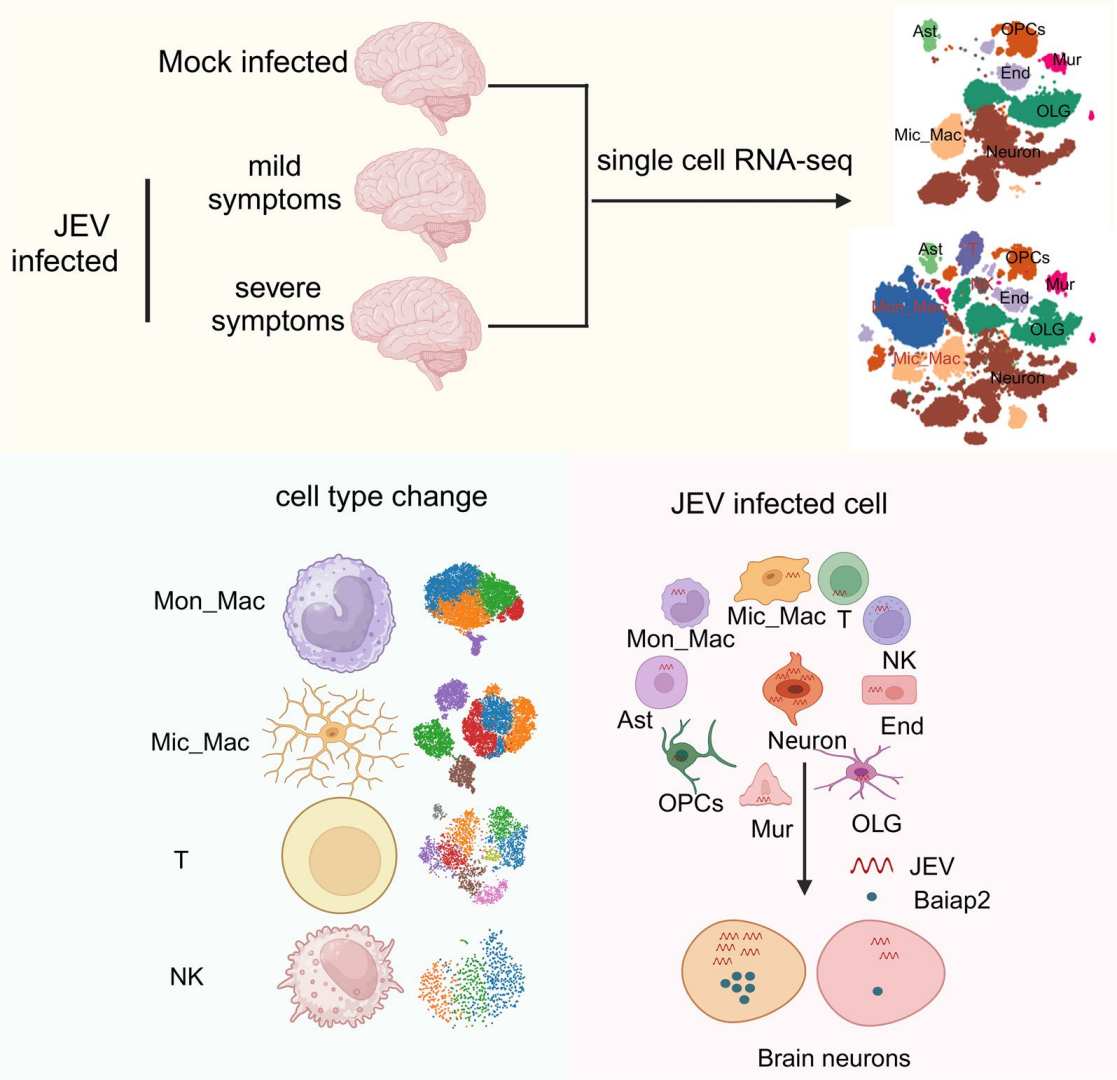
Overall workflow and descriptions of each step of the study. The diagram was constructed with BioRender (https://biorender.com/)

Currently, there are limited therapeutic drugs and methods available for treating viral encephalitis, primarily due to the complex pathogenesis in the CNS. In this study, we employed scRNA-seq to generate a comprehensive landscape of cellular composition and immunological phenotype in the brain tissue of mice infected with JEV. Our findings revealed profound immune activation, with significant increases in the numbers of Mon_Mac, T cells, NK cells, as well as activated Mic_Mac. We detected JEV genes in almost all types of brain cells, but their expression was predominantly found in neuronal cells, particularly in Baiap2 + neurons. As the symptoms worsened, there was a notable escalation in both the infiltration of immune cells and the damage caused to neurons, accompanied by a gradual rise in the number of JEV-infected cells. These findings make significant contributions to our comprehension of the pathogenesis of JEV and other viral encephalitis (Fig. 8).

IHC analysis revealed that JEV was primarily distributed in the cerebral cortex, striatum, and thalamus of the mice [29]. This distribution could be attributed to the enrichment of neurons in these regions [47]. On the other hand, the cerebral cortex has a high density of blood vessels, making the cells in this region more susceptible to JEV infection once it breaches the blood–brain barrier.

By analyzing the results of scRNA-Seq on cells isolated from JEV-infected regions in mouse brains, we found the proportion of neurons did not show significant changes in the brains of mice with mild symptoms; however, there was a substantial decrease in the proportion of neurons in the brains of mice with severe symptoms, compared to mock-infected mice. This finding supports the notion that the development of JEV-caused diseases is closely correlated with neuronal damage. Additionally, our findings indicate a significant decrease in the proportion of astrocyte, oligodendrocytes and oligodendrocyte precursor cells as symptoms worsen. On one hand, JEV infection could lead to extensive cell death. On the other hand, the infiltration of immune cells results in an increase in total number of cell types in the mouse brain, thereby may reduce the relative ratio of glial cells. However, the percentage of Mic_Mac, which are resident immune cells that initiate inflammation and immune response in the brain, did not change significantly. Instead, they transitioned from being Tmem119 positive to Aif1 positive, indicating a shift from a resting state to an activated state [48, 49]. Subclass analysis of Mic_Mac revealed the emergence of three distinct groups of cells after viral infection. One of a subcluster consisted of cells from the mild symptomatic mice, which not only expressed the resting state marker but also expressed Il1b and Tlr3 specifically. The other two groups comprised cells from the severely symptomatic mice, which specifically expressed Itgax and Ly6c2, indicating that these two groups of cells are activated microglia that play a role in antigen-presenting [33]. However, further research is required to understand the transition of microglia from a resting state to an activated state, as well as how they recruit immune cells to exert antiviral effects under different symptoms.

Furthermore, we observed a decrease in the proportion of endothelial cells in JEV-infected mouse brain, which is associated with the disruption of blood–brain barrier during JEV infection [50–52]. However, there were no significant changes in the number of mural cells, which include pericytes and vascular smooth muscle cells and play a significant role in maintaining the integrity of the vascular wall. Previous studies have reported that direct communication between mural cells and tumor cells promotes cancer growth [53]. In our study, we identified a significant number of mural cells infected with JEV. Analysis of differentially regulated genes in mural cells after JEV infection revealed an up-regulation of antiviral genes, suggesting that mural cells may contribute to the resistance against JEV infection.

In addition, our study also revealed a strong correlation between the intensity of symptoms and the infiltration of immune cells. Specifically, we observed a gradual increase in the proportions of Mon_Mac, NK cells, and T cells as the severity of symptoms escalated. It is worth to noting that B cells and neutrophils were not detected using scRNA-seq, despite previous reports of increased presence in the brain after JEV and other encephalitis virus infection [54–57]. This discrepancy could be attributed to the use of Miltenyi's Debris Removal Solution (130-109-398) during the preparation of the single-cell suspension for scRNA-seq. In order to effectively isolate neural cells and remove cell fragments from brain tissues, the debris removal reagent from Miltenyi was employed for single-cell isolation prior to RNA sequencing and flow cytometry. However, this reagent may unintentionally remove B cells and neutrophils, which are relatively smaller in size. As a result, we were unable to detect these two types of cells in this study.

Consistent with previous studies, our scRNA-seq results demonstrated that the JEV genome is predominantly found in neurons [58, 59]. Moreover, as the symptoms worsened, the abundance of viral genes increased. According to the recent research, SARS-CoV-2 has been found to have a preference for dopaminergic neurons [60]. To determine whether JEV also exhibits a tropism for specific types of neurons based on the neurotransmitters they secrete, we analyzed the susceptibility of glutamatergic, aminobutyric acid, dopaminergic, serotoninergic, and cholinergic neurons to JEV. Our analysis revealed that both glutamatergic and aminobutyric acid neurons were highly susceptible to JEV infection (Additional file 10: Fig. S10). However, dopaminergic and serotoninergic neurons were not detected in the scRNA-seq results. This may be attributed to the fact that the brain regions selected for scRNA-seq were the cerebral cortex, striatum, and thalamus, while dopaminergic neurons are primarily distributed in the substantia nigra of the midbrain, and serotoninergic neurons are mainly located in the pineal gland and hypothalamus. In addition, only a small number of cholinergic neurons were detected, which may be attributed to their main distribution in the striatum but not the cerebral and thalamus. However, there were notable variations in the expression levels of viral genes among different clusters of neuronal cells, indicating a preference of JEV for specific neurons. Furthermore, this study provides evidence that the viral susceptibility of neurons is correlated to the expression of Baiap2. This finding was further confirmed by IHC and FISH assays. Baiap2 is protein that specifically inhibits the growth of blood vessels in the brain. It acts as a bridge between G proteins on the cell membrane and cytoplasmic effector proteins. Baiap2 as an important component of the postsynaptic density (PSD) and excitatory synapses. In spiny excitatory neurons of the neocortex and hippocampus, Baiap2 is concentrated in the center of the PSD, whereas in spiny inhibitory neurons of the neostriatum and cerebellar cortex, it is evenly distributed along the lateral axis of the PSD [61]. Recent studies have associated IRSp53/Baiap2 with various psychiatric disorders, including autism spectrum disorder (ASD) [57], attention-deficit/hyperactivity disorder (ADHD) [58] and schizophrenia [59]. According to the results of in situ hybridization assay from the Allen Brain Atlas website (https://mouse.brain-map.org/), Baiap2 is primarily found in the cerebral cortex and striatum, which have been identified as JEV-susceptible regions in the brain (Additional file 1: Fig. S1E, F). Previous studies have indicated that mice lacking Baiap2 exhibit enhanced activation of the N-methyl-D-aspartate receptor (NMDAR), resulting in social and cognitive deficits [62, 63]. Furthermore, the activation of NMDAR has been linked to increased neuronal damage during JEV infection [64]. These findings suggest that high expression of Baiap2 may inhibit NMDAR activation, thereby preventing neuronal death and inflammatory response caused by JEV infection and promoting viral propagation. However, the specific mechanism by which Baiap2 affects JEV replication requires further investigation.

Infection with JEV can lead to a variety of symptoms in different individuals. However, the underlying mechanism behind these differences remains unclear. Our study also showed different symptoms in mice after JEV infection. By comparing the cell types and viral distribution in mice with mild and severe symptoms, it is speculated that the variation in symptoms may be attributed to several factors. These include the disruption of the blood–brain barrier, neuronal damage, activation of microglia, and the infiltration of inflammation-related cells. This was evidenced by noticeable difference in the presence of neurons, activated microglia, and infiltrating immune cells, as well as the expression of genes related to tight junctions in endothelial cells, inflammatory cytokines, chemokines and antiviral response in Mic_Mon, Mon_Mac, NK and T cells, between mice with mild and severe symptoms. Following JEV infection, the virus initially proliferates in the blood and peripheral organs. Once it breaches the blood–brain barrier, it extensively multiplies in neurons, leading to neuronal damage and microglial activation [65]. The activated microglia release inflammatory cytokines and chemokines, which in turn cause the infiltration of inflammatory cells [66]. This exacerbates neuronal damage in the CNS. While our study sheds light on the potential mechanisms underlying the development of different symptoms in mice after JEV infection, further investigation is needed to fully understand the individual differences in JEV-induced clinical symptoms.

In summary, by conducting a scRNA-seq analysis on the brain tissues of JEV-infected mice, our study revealed a decrease in number of neurons and an increased presence of activated microglia and infiltrating immune cells, including monocytes and macrophages, T cells, and NK cells. These cells were found to be associated with the severity of symptoms in JEV-infected mice. We also observed enhanced communication between individual cells and significant ligand-receptor pairs related to tight junctions, chemokines, and antigen-presenting molecules following JEV infection, suggesting an upregulation of endothelial permeability and inflammatory response in response to JEV infection. Furthermore, our research identified Baiap2-positive neurons as particularly susceptible to JEV. These findings provide valuable insights into the mechanism of JEV-induced neuro-damage and inflammation, as well as potential avenues for developing therapies for Japanese encephalitis.

### Supplementary Information

**Additional file 1: Figure S1.** Clinical signs of mice with JEV infection and the distribution of JEV in the mouse brain. (A) Mice body weight was monitored every day after infection. (B) Clinical symptoms were record after 7 days post-infection. (C) Specific clinical signs in mild symptoms and severe symptoms. (D) Virus titers in mouse brains (n = 3) were determined by plaque forming assay. (E, F) The distribution of JEV E protein in the brain tissues of mice with mild symptom and severe symptom was detected by immunohistochemical. (E) Sagittal slice, (F) coronal slice. (section = 5 μm, scale bars = 2 mm or 100 μm). Data from 3 slices were presented as mean ± SEM. The statistics were analyzed using two-tailed Student’s t-test. **P < 0.01, ns, nonsignificant.

**Additional file 2: Figure S2.** Preparation of single cell suspension. (A) Body weight and clinical symptoms of mice used for scRNA-seq. (B) Brain slices for preparing the single cell suspension. Five-week-old C57/BL6 mice were intraperitoneal injected with 10^5^ PFU JEV. And mock-infected group were injected with DMEM. 7 days after infection, mock-infected mice and JEV-infected mice with different symptoms cardiac perfusion with CCS. The brain tissue is sliced on a vibration slicer, 300 μm per slice. The brain slice located in the red box were selected and separated of the cortex, striatum, and thalamus using a microscope to prepare a single cell suspension. (C) Different cell morphologies of single-cell suspension. (D) The cell viability was determined by flow cytometry. (E) JEV RNA copy numbers in cell suspensions were measured by qPCR. Data from 3 independent experiments were presented as mean ± SEM. The statistics were analyzed using two-tailed Student’s t-test. **P < 0.01.

**Additional file 3: Figure S3.** Single-cell transcriptional profiling of the brain tissues of mice with JEV infection. (A) Overview of the clusters in the integrated single-cell transcriptome. (B) Heatmap visualization of cluster marker expression. (C, D) Proportion of different clusters (C) and different cell types (D) in brains of mock- and JEV-infected mice. E t-SNE visualization of the brain cells in mock- and JEV-infected mice.

**Additional file 4: Figure S4.** Immunohistochemistry analysis of brain tissues of JEV-infected and mock-infected mice. Immunohistochemistry images of Iba1, Ly6c, CD49b, CD8a+ T and CD4+ T cells in the brains of mock- and JEV-infected mice. The scale bar is 200 μm.

**Additional file 5: Figure S5.** Analysis of differentially regulated genes and their functions in different cell types. (A–I) Volcano plot of differentially expressed gene in Ast (A), End (B), Mic_Mac (C), Mur (D), NK (E), OPCs (F), OLG (G), T (H) and NeuN (I) in mouse brain after JEV infection, and functional analysis of the differentially regulated genes.

**Additional file 6: Figure S6.** Subclustering of Mic_Mon and the potential developmental trajectory of cell subsets. (A) tSNE visualization of clustering revealed six distinct Mic_Mon cell populations. Each dot on the plot represents a single cell and is colored according to its subset. (B) Histogram showing the proportions of cells at each group and in each subcluster. (C) Heatmap of the top marker genes per cluster. (D, E) Pseudotime trajectory analysis across all time points using single cells from mock-infected, mild symptom, and severe symptom groups. The visualization depicted cell trajectories of cluster (D) and the pseudotime (E). (F) Heatmap revealed pseudotemporal expression patterns of clustering genes, with cells (column) are ordered according to the pseudotime development.

**Additional file 7: Figure S7.** Subclustering of Mon_Mac cells and potential developmental trajectory of Mon_Mac cell subsets. (A) tSNE visualization of clustering revealed six distinct Mon_Mac cell populations. Each dot represents a single cell and was colored according to subsets. (B) Histogram showing the proportions of cells in each group and subcluster. (C) tSNE plot of Mic_Mon cell-specific markers and genes specifically expressed in different subclusters. (D, E) Pseudotime trajectory analysis across all time points with single cells from mock-infected mice and JEV-infected mice with mild symptom or moderate symptom. Visualization of cell trajectories of cluster (D) and the pseudotime (E). (F) Heatmap revealed pseudotemporal expression patterns of clustering genes. Cells (column) are ordered according to the pseudotime development.

**Additional file 8: Figure S8.** Subclustering of T cells and potential developmental trajectory of T cell subsets. (A) tSNE visualization of clustering revealed nine distinct T cell populations. Each dot represents a single cell and was colored according to subsets. (B) Histogram showing the proportions of cells in each group and subcluster. (C) tSNE plot of T cell-specific markers and genes specifically expressed in different subclusters. (D, E) Pseudotime trajectory analysis across all time points using single cells from mock-infected mice and JEV-infected mice with mild symptom or moderate symptom. Visualization of cell trajectories of cluster (D) and the pseudotime (E). (F) Heatmap revealed pseudotemporal expression patterns of clustering genes. Cells (column) are ordered according to the pseudotime development.

**Additional file 9: Figure S9.** Subclustering of NK cells and potential developmental trajectory of NK cell subsets. (A) tSNE visualization of clustering revealed 3 distinct NK cell populations. Each dot represents a single cell and was colored according to subsets. (B) Histogram showing the proportions of cells in each group and subcluster. (C) tSNE plot of NK cell-specific markers and genes specifically expressed in different subclusters. (D, E) Visualization of cell trajectories of cluster (D) and the pseudotime (E). (F) Heatmap revealed pseudotemporal expression pattern of clustering genes. Cells (column) are ordered according to the pseudotime development.

**Additional file 10: Figure S10.** Distribution of JEV in different neuronal types based on the secretion of neurotransmitters. (A) Classification of different neuronal types according to their secretion of neurotransmitters. (B) Heatmap showing the expressions of Slc17a6 and Slc17a7 (glutamatergic neuron), Gad1 (aminobutyric acid neurons), Th (dopaminergic neurons), Tph2 (serotoninergic neurons), and Chat (cholinergic neuron). (C) Expression of JEV genes in different neuronal types. (D) Percentages of JEV-positive cells to total neuron cells in different neuronal types.

## Data Availability

The accession number for the scRNA-seq datasets reported in this paper is GEO: GSE237915. This paper does not report original code. Any additional information required to reanalyze the data reported in this work paper is available from the lead contact upon request.

## Abbreviations

scRNA-seq: Single-cell RNA sequencing
JEV: Japanese encephalitis virus
CNS: Central nervous system
DMEM: Dulbecco’s modified Eagle’s medium
dpi: Days post infection
IHC: Immunohistochemistry
FISH: Fluorescence in situ hybridization
UMI: Unique molecular identifier
PFU: Plaque forming units
