# Supplementary material for: Single-cell RNA sequencing reveals the immune features and viral tropism in the central nervous system of mice infected with Japanese encephalitis virus
Source: J Neuroinflammation. 2024 Mar 26;21:76. doi: 10.1186/s12974-024-03071-1 (PMC10967088; doi:10.1186/s12974-024-03071-1)

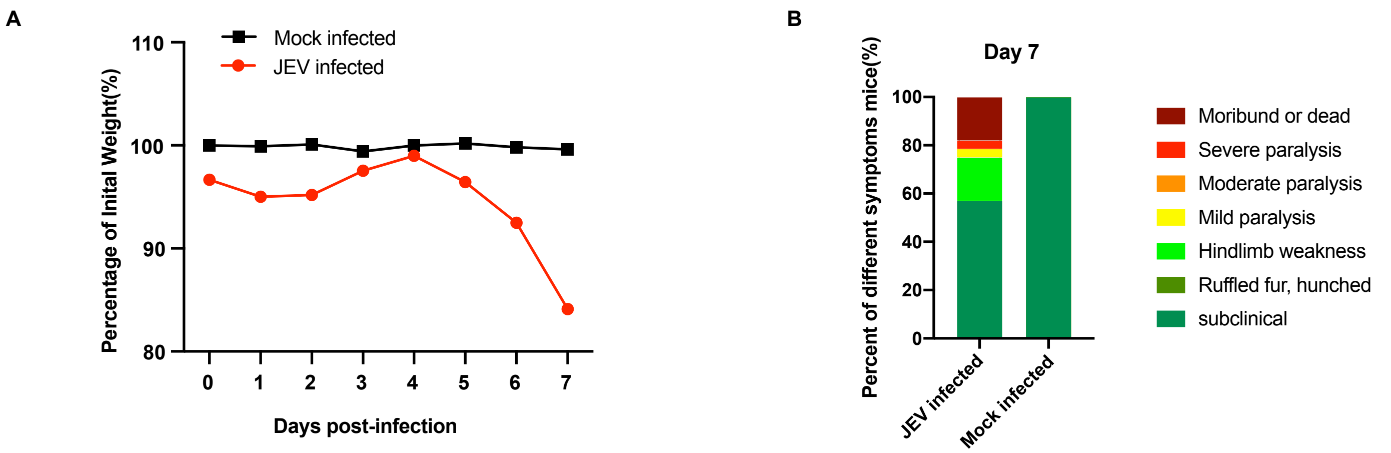

C

Group	Symptoms
Mock infected	subclinical
Mild symptoms	ruffled fur, hindlimb weakness
Severe symptoms	quiver, paralysis

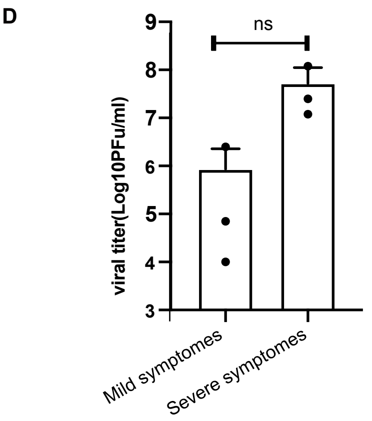

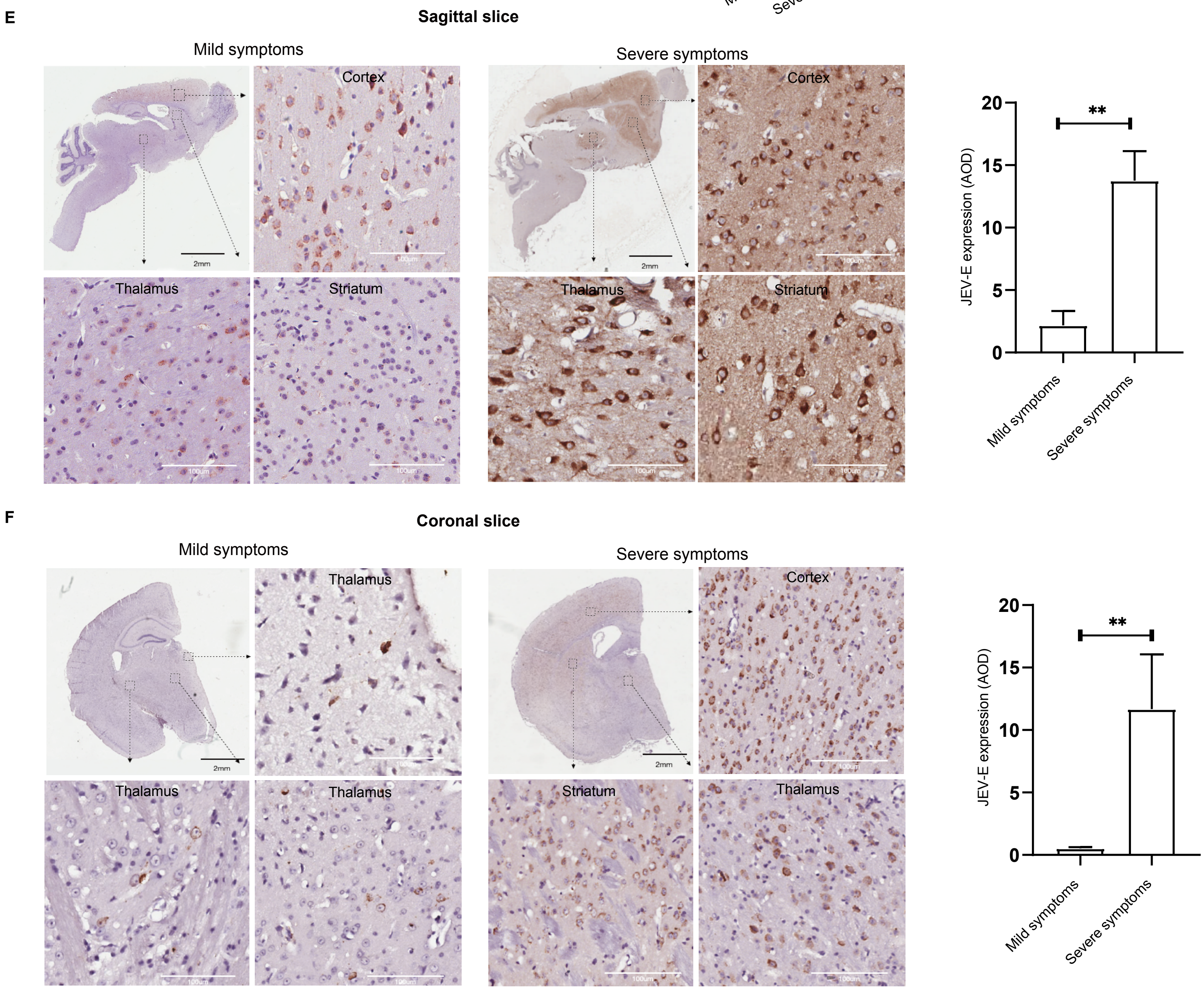

Supplement: Supplementary file 1 — Additional file 1: Figure S1. Clinical signs of mice with JEV infection and the distribution of JEV in the mouse brain. (A) Mice body weight was monitored every day after infection. (B) Clinical symptoms were record after 7 days post-infection. (C) Specific clinical signs in mild symptoms and severe symptoms. (D) Virus titers in mouse brains (n = 3) were determined by plaque forming assay. (E, F) The distribution of JEV E protein in the brain tissues of mice with mild symptom and severe symptom was detected by immunohistochemical. (E) Sagittal slice, (F) coronal slice. (section = 5 μm, scale bars = 2 mm or 100 μm). Data from 3 slices were presented as mean ± SEM. The statistics were analyzed using two-tailed Student’s t-test. **P < 0.01, ns, nonsignificant. [file 12974_2024_3071_MOESM1_ESM.pdf]
