# Supplementary figures and images for: Single-cell RNA sequencing reveals the immune features and viral tropism in the central nervous system of mice infected with Japanese encephalitis virus
Source: J Neuroinflammation. 2024 Mar 26;21:76. doi: 10.1186/s12974-024-03071-1 (PMC10967088; doi:10.1186/s12974-024-03071-1)

**A**

| Group           | Weight(g) |
|-----------------|-----------|
| Mock infected   | 17.1-17.6 |
| Mild symptoms   | 13.4-14.5 |
| Severe symptoms | 12.8-13.5 |

**B**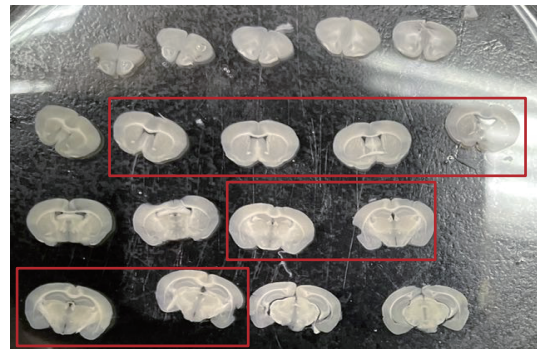**C**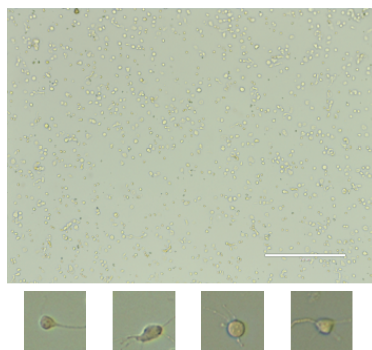**D**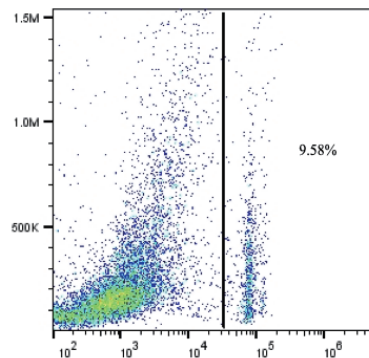**E**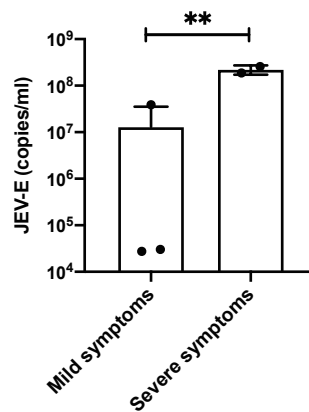

Supplement: Supplementary file 2 — Additional file 2: Figure S2. Preparation of single cell suspension. (A) Body weight and clinical symptoms of mice used for scRNA-seq. (B) Brain slices for preparing the single cell suspension. Five-week-old C57/BL6 mice were intraperitoneal injected with 105 PFU JEV. And mock-infected group were injected with DMEM. 7 days after infection, mock-infected mice and JEV-infected mice with different symptoms cardiac perfusion with CCS. The brain tissue is sliced on a vibration slicer, 300 μm per slice. The brain slice located in the red box were selected and separated of the cortex, striatum, and thalamus using a microscope to prepare a single cell suspension. (C) Different cell morphologies of single-cell suspension. (D) The cell viability was determined by flow cytometry. (E) JEV RNA copy numbers in cell suspensions were measured by qPCR. Data from 3 independent experiments were presented as mean ± SEM. The statistics were analyzed using two-tailed Student’s t-test. **P < 0.01. [file 12974_2024_3071_MOESM2_ESM.pdf]

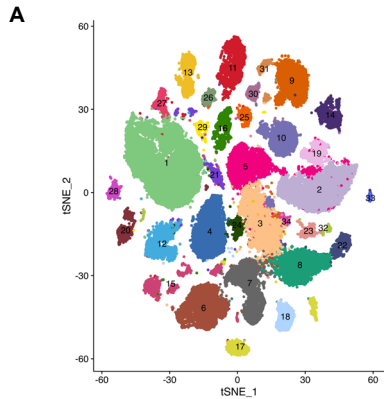

**B**

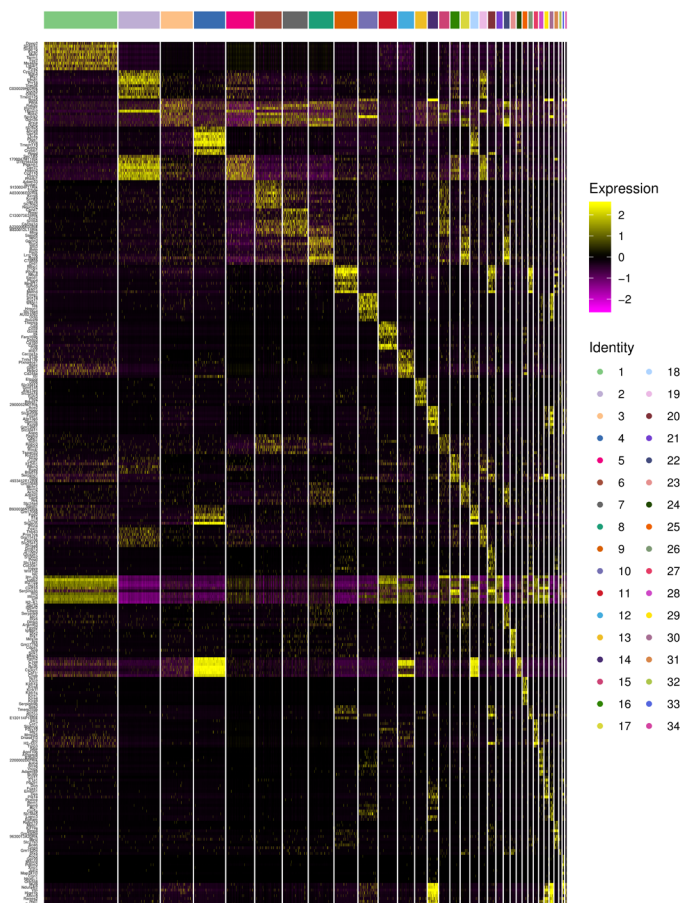

**C**

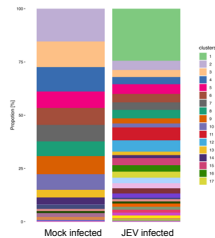

**D**

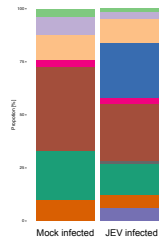

**E**

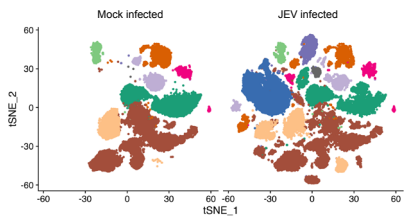

Supplement: Supplementary file 3 — Additional file 3: Figure S3. Single-cell transcriptional profiling of the brain tissues of mice with JEV infection. (A) Overview of the clusters in the integrated single-cell transcriptome. (B) Heatmap visualization of cluster marker expression. (C, D) Proportion of different clusters (C) and different cell types (D) in brains of mock- and JEV-infected mice. E t-SNE visualization of the brain cells in mock- and JEV-infected mice. [file 12974_2024_3071_MOESM3_ESM.pdf]

Mock infected

JEV infected

Iba1

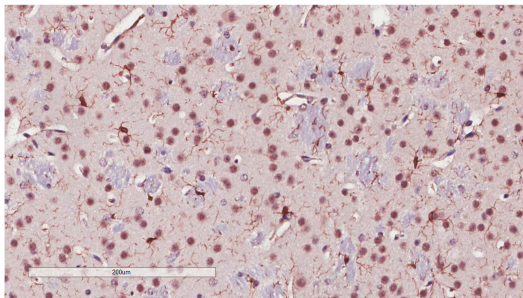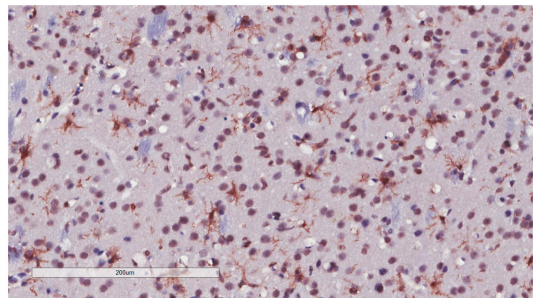

Ly6c

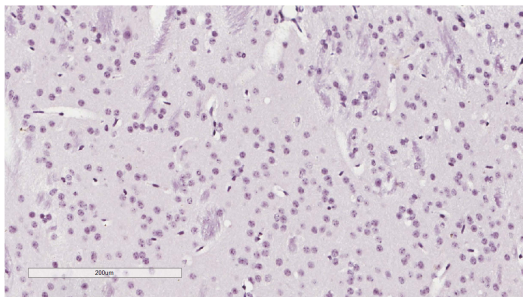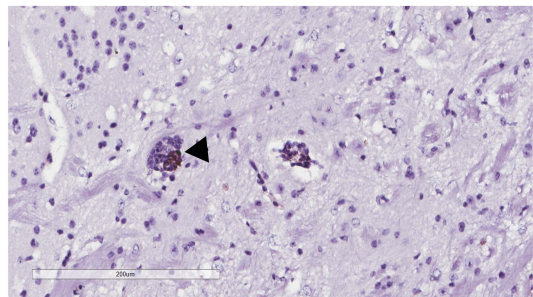

CD49b

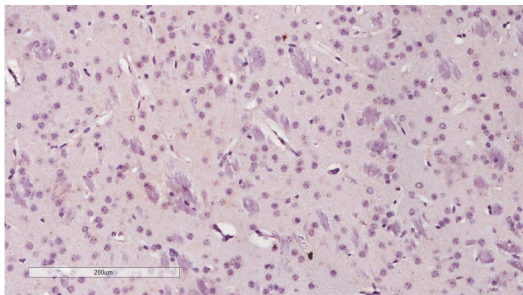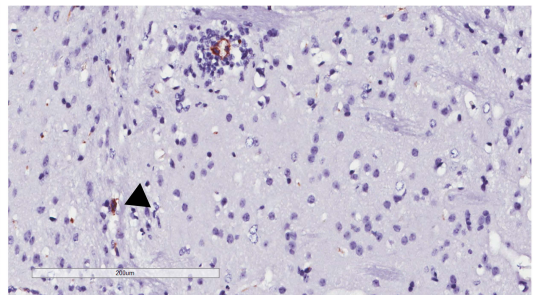

CD8a

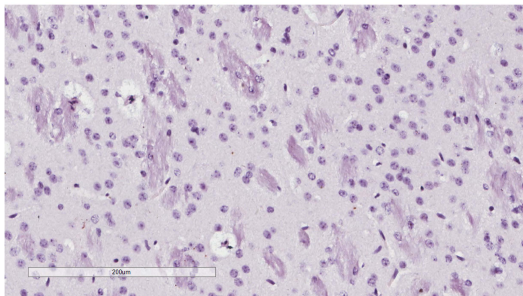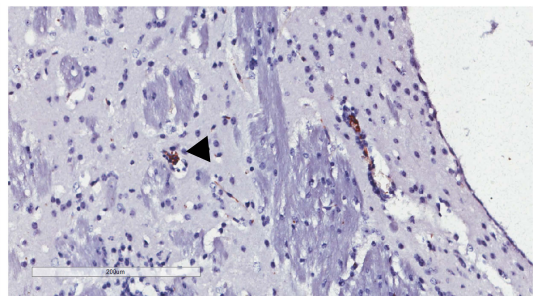

CD4

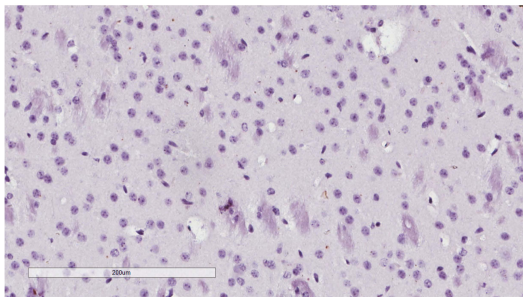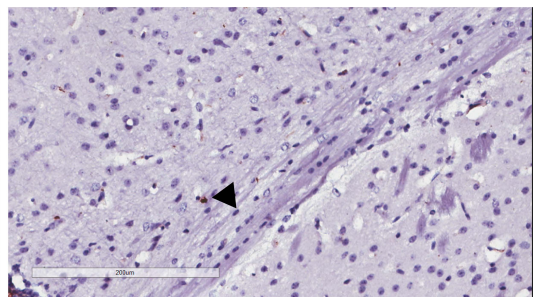

Supplement: Supplementary file 4 — Additional file 4: Figure S4. Immunohistochemistry analysis of brain tissues of JEV-infected and mock-infected mice. Immunohistochemistry images of Iba1, Ly6c, CD49b, CD8a+ T and CD4+ T cells in the brains of mock- and JEV-infected mice. The scale bar is 200 μm. [file 12974_2024_3071_MOESM4_ESM.pdf]

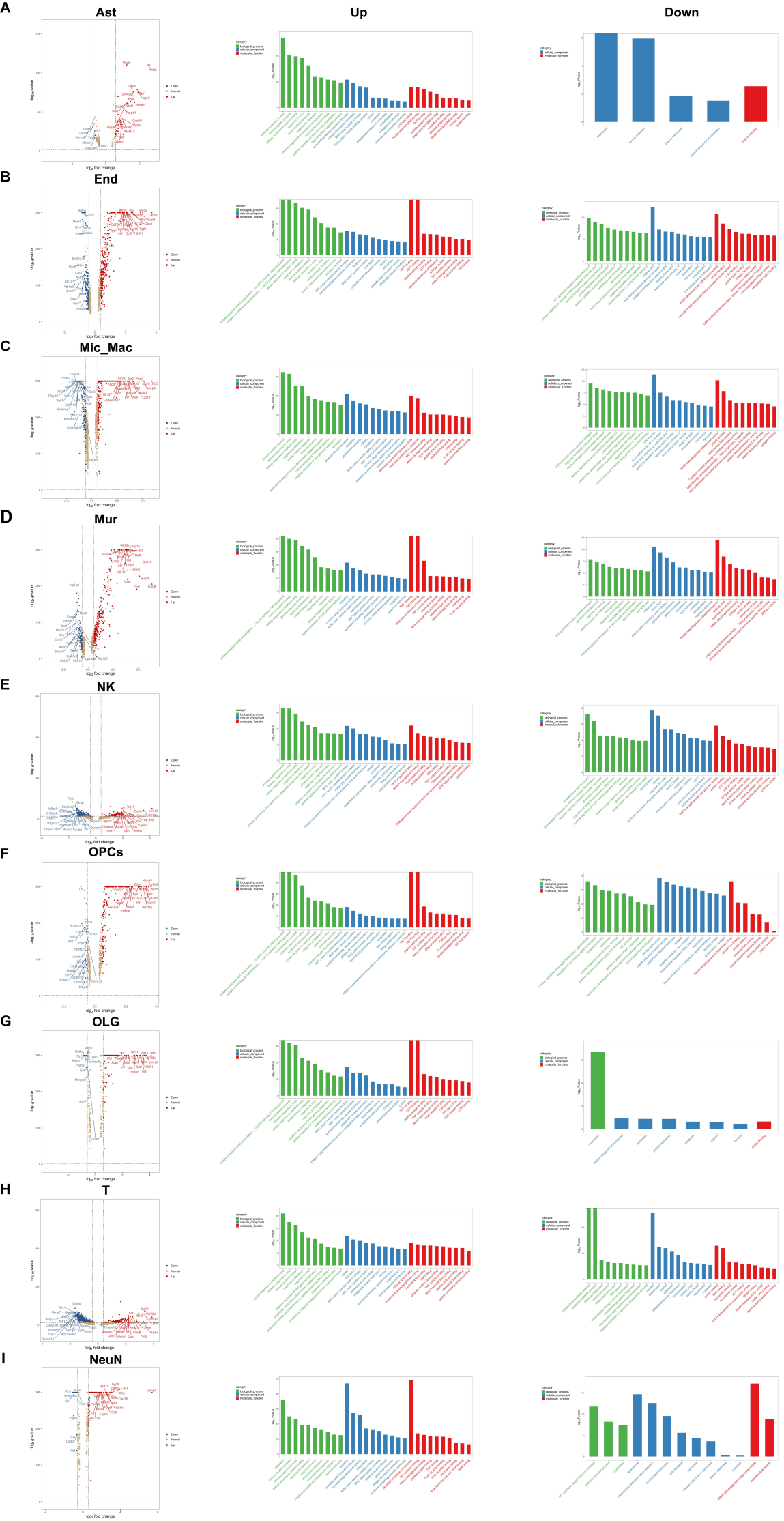

Supplement: Supplementary file 5 — Additional file 5: Figure S5. Analysis of differentially regulated genes and their functions in different cell types. (A–I) Volcano plot of differentially expressed gene in Ast (A), End (B), Mic_Mac (C), Mur (D), NK (E), OPCs (F), OLG (G), T (H) and NeuN (I) in mouse brain after JEV infection, and functional analysis of the differentially regulated genes. [file 12974_2024_3071_MOESM5_ESM.pdf]

A

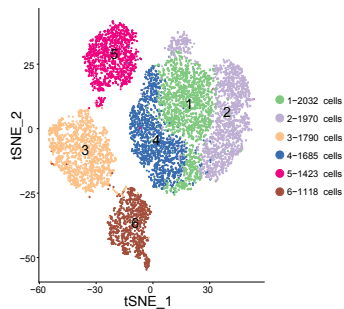

B

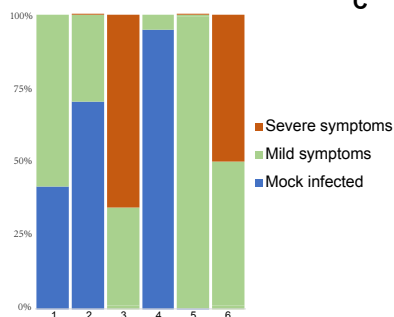

C

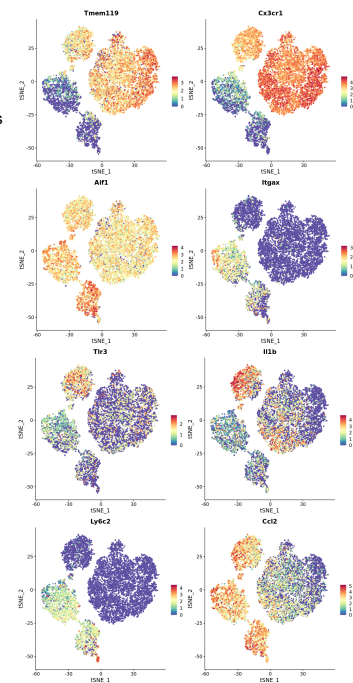

D

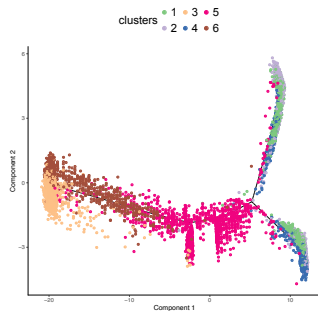

E

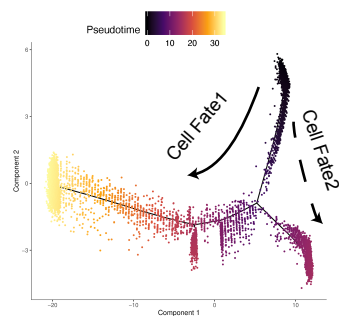

F

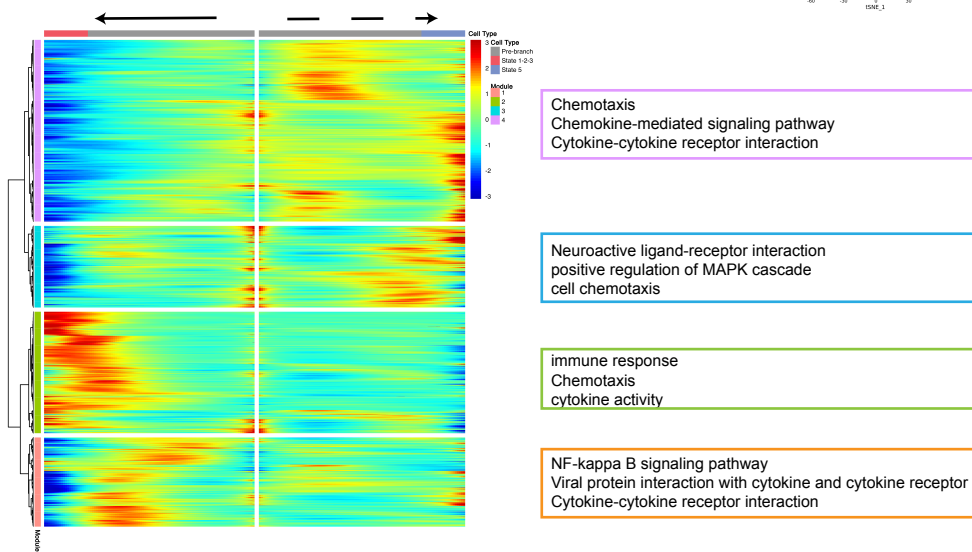

Supplement: Supplementary file 6 — Additional file 6: Figure S6. Subclustering of Mic_Mon and the potential developmental trajectory of cell subsets. (A) tSNE visualization of clustering revealed six distinct Mic_Mon cell populations. Each dot on the plot represents a single cell and is colored according to its subset. (B) Histogram showing the proportions of cells at each group and in each subcluster. (C) Heatmap of the top marker genes per cluster. (D, E) Pseudotime trajectory analysis across all time points using single cells from mock-infected, mild symptom, and severe symptom groups. The visualization depicted cell trajectories of cluster (D) and the pseudotime (E). (F) Heatmap revealed pseudotemporal expression patterns of clustering genes, with cells (column) are ordered according to the pseudotime development. [file 12974_2024_3071_MOESM6_ESM.pdf]

**A**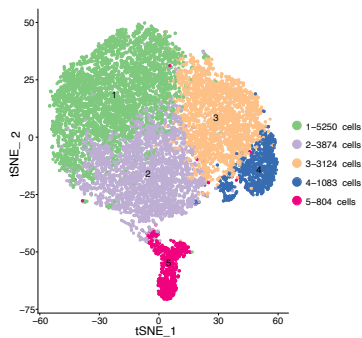**B**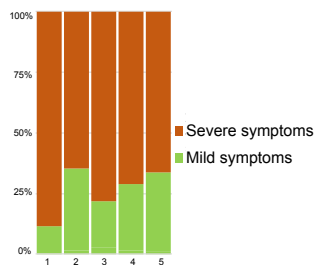**C**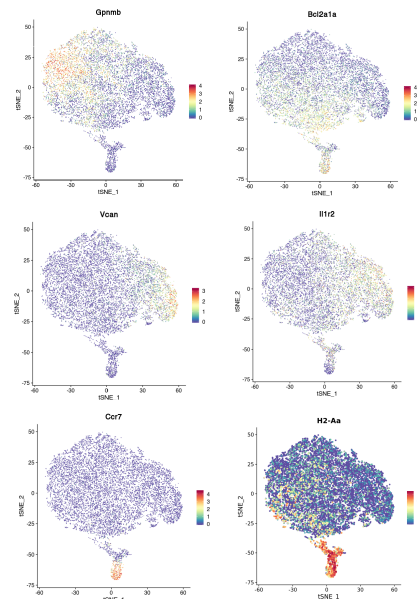**D**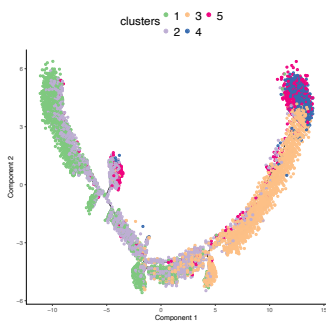**E**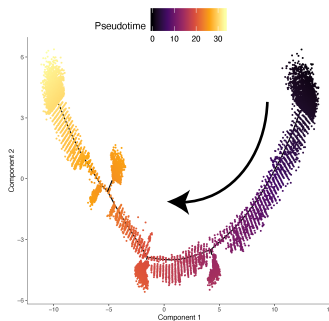**F**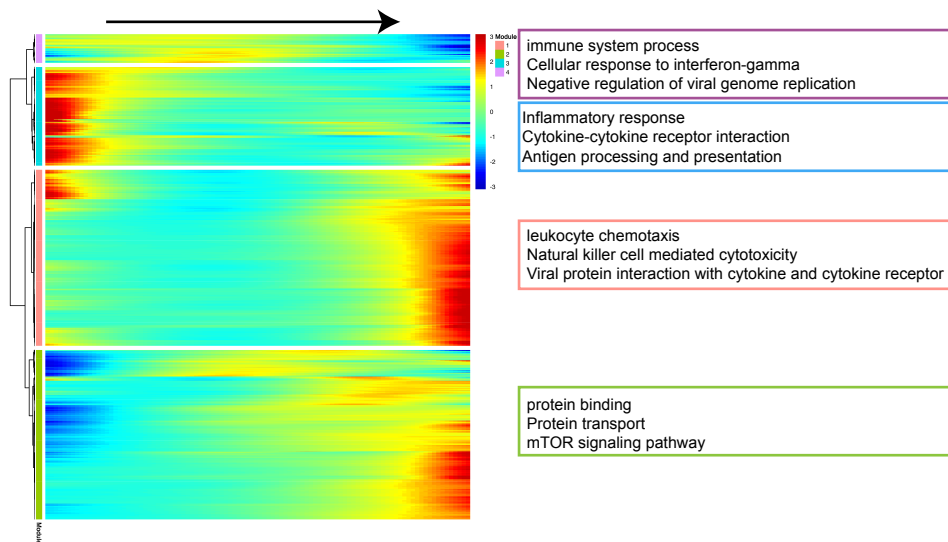

Supplement: Supplementary file 7 — Additional file 7: Figure S7. Subclustering of Mon_Mac cells and potential developmental trajectory of Mon_Mac cell subsets. (A) tSNE visualization of clustering revealed six distinct Mon_Mac cell populations. Each dot represents a single cell and was colored according to subsets. (B) Histogram showing the proportions of cells in each group and subcluster. (C) tSNE plot of Mic_Mon cell-specific markers and genes specifically expressed in different subclusters. (D, E) Pseudotime trajectory analysis across all time points with single cells from mock-infected mice and JEV-infected mice with mild symptom or moderate symptom. Visualization of cell trajectories of cluster (D) and the pseudotime (E). (F) Heatmap revealed pseudotemporal expression patterns of clustering genes. Cells (column) are ordered according to the pseudotime development. [file 12974_2024_3071_MOESM7_ESM.pdf]

A

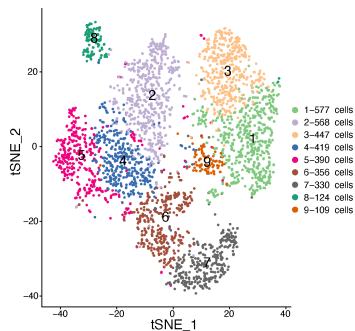

B

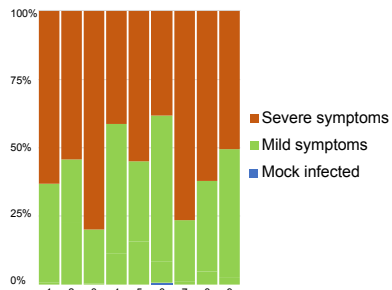

C

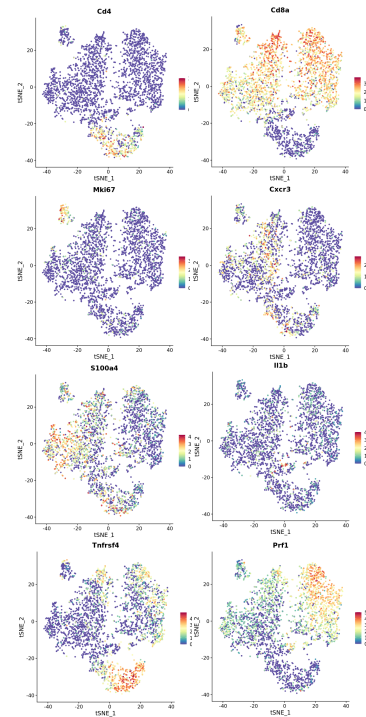

D

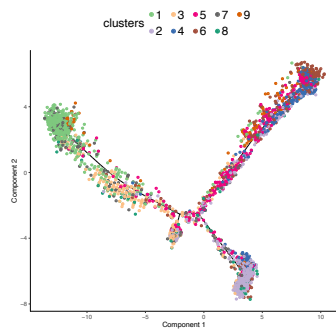

E

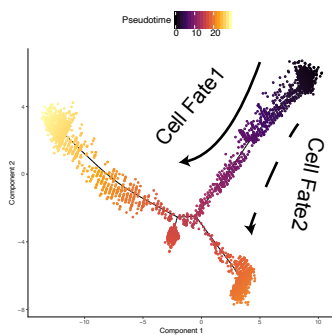

F

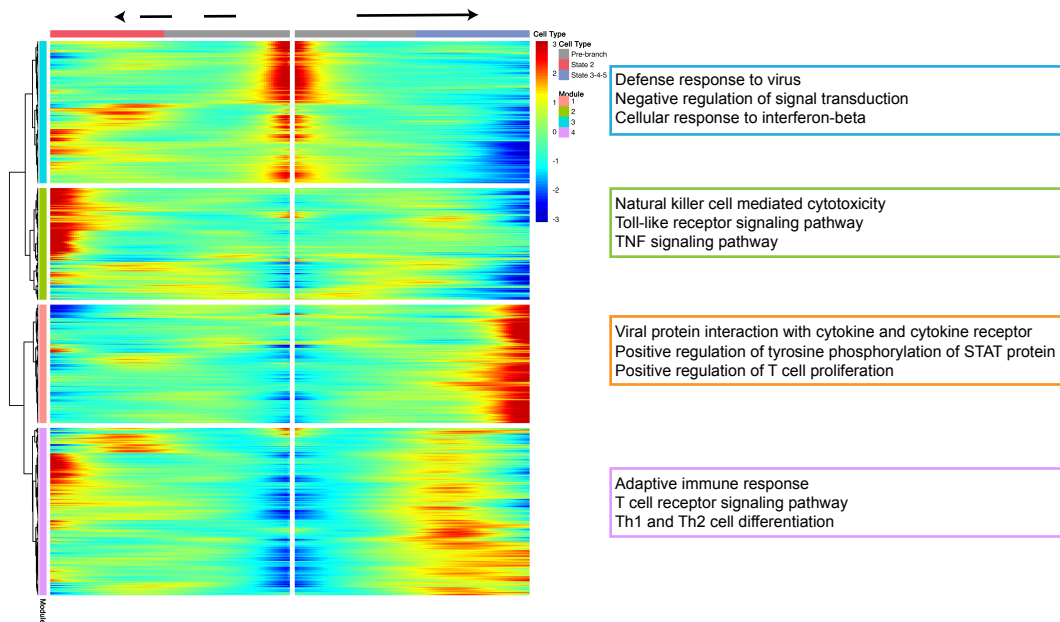

Supplement: Supplementary file 8 — Additional file 8: Figure S8. Subclustering of T cells and potential developmental trajectory of T cell subsets. (A) tSNE visualization of clustering revealed nine distinct T cell populations. Each dot represents a single cell and was colored according to subsets. (B) Histogram showing the proportions of cells in each group and subcluster. (C) tSNE plot of T cell-specific markers and genes specifically expressed in different subclusters. (D, E) Pseudotime trajectory analysis across all time points using single cells from mock-infected mice and JEV-infected mice with mild symptom or moderate symptom. Visualization of cell trajectories of cluster (D) and the pseudotime (E). (F) Heatmap revealed pseudotemporal expression patterns of clustering genes. Cells (column) are ordered according to the pseudotime development. [file 12974_2024_3071_MOESM8_ESM.pdf]

A

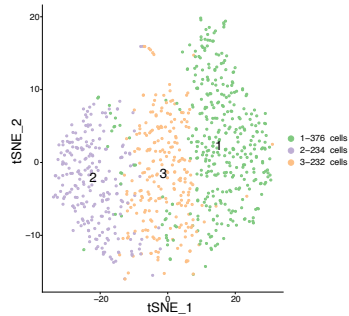

B

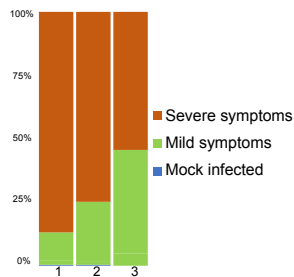

C

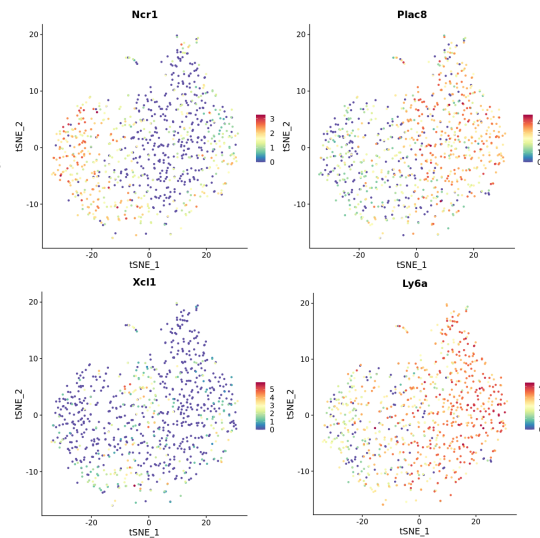

D

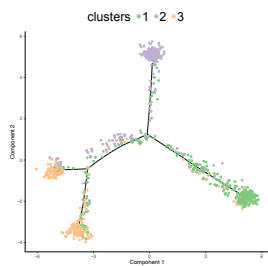

E

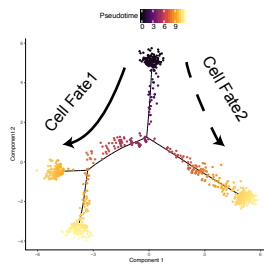

F

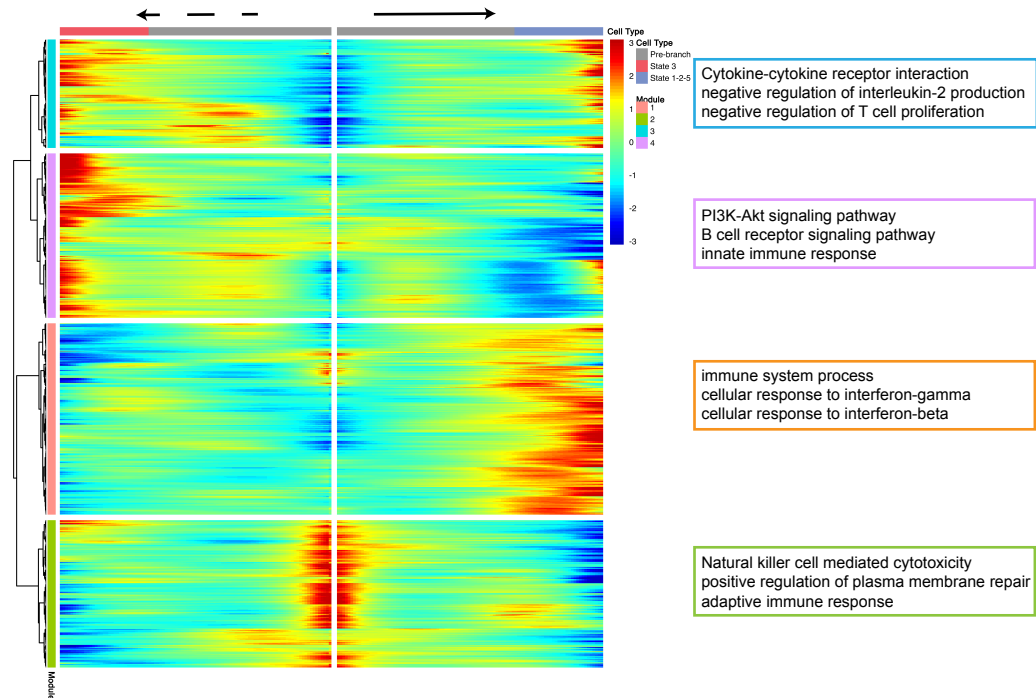

Supplement: Supplementary file 9 — Additional file 9: Figure S9. Subclustering of NK cells and potential developmental trajectory of NK cell subsets. (A) tSNE visualization of clustering revealed 3 distinct NK cell populations. Each dot represents a single cell and was colored according to subsets. (B) Histogram showing the proportions of cells in each group and subcluster. (C) tSNE plot of NK cell-specific markers and genes specifically expressed in different subclusters. (D, E) Visualization of cell trajectories of cluster (D) and the pseudotime (E). (F) Heatmap revealed pseudotemporal expression pattern of clustering genes. Cells (column) are ordered according to the pseudotime development. [file 12974_2024_3071_MOESM9_ESM.pdf]

A

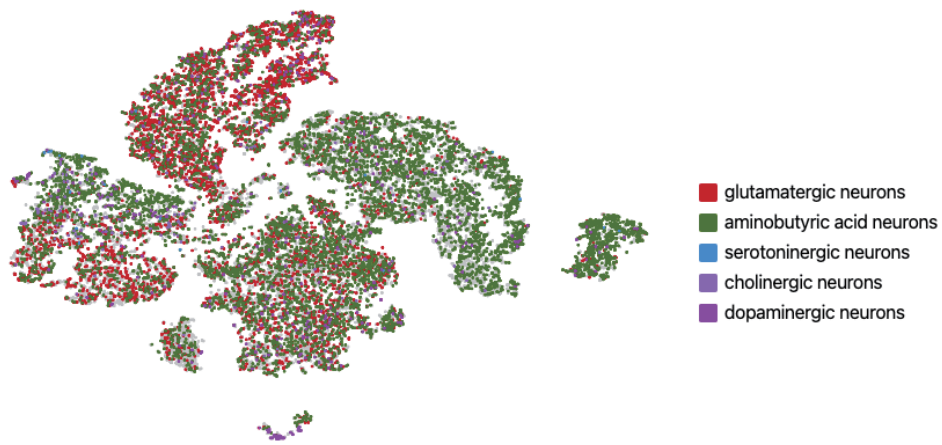

B

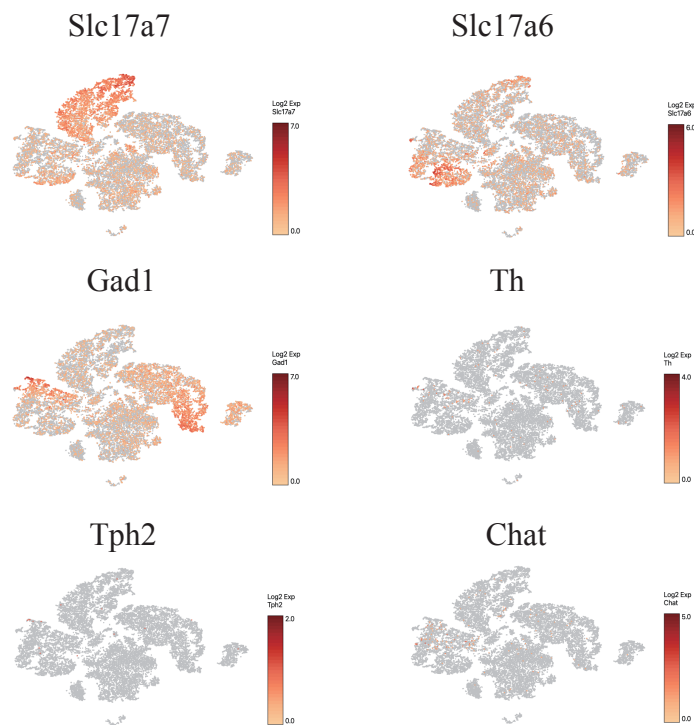

C

JEV

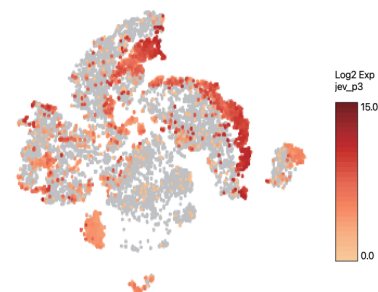

D

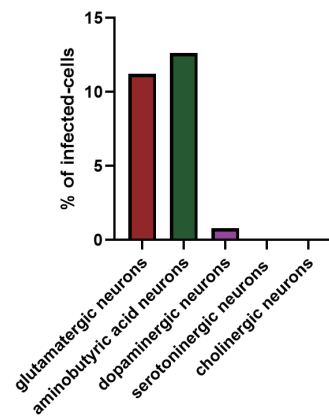

Supplement: Supplementary file 10 — Additional file 10: Figure S10. Distribution of JEV in different neuronal types based on the secretion of neurotransmitters. (A) Classification of different neuronal types according to their secretion of neurotransmitters. (B) Heatmap showing the expressions of Slc17a6 and Slc17a7 (glutamatergic neuron), Gad1 (aminobutyric acid neurons), Th (dopaminergic neurons), Tph2 (serotoninergic neurons), and Chat (cholinergic neuron). (C) Expression of JEV genes in different neuronal types. (D) Percentages of JEV-positive cells to total neuron cells in different neuronal types. [file 12974_2024_3071_MOESM10_ESM.pdf]
